# Exploring the intersection of obesity and gender in COVID-19 outcomes in hospitalized Mexican patients: a comparative analysis of risk profiles using unsupervised machine learning

**DOI:** 10.3389/fpubh.2024.1337432

**Published:** 2024-04-18

**Authors:** Fahimeh Nezhadmoghadam, José Gerardo Tamez-Peña, Emmanuel Martinez-Ledesma

**Affiliations:** ^1^Tecnologico de Monterrey, The Institute for Obesity Research, Monterrey, Mexico; ^2^Tecnologico de Monterrey, Escuela de Medicina y Ciencias de la Salud, Monterrey, Mexico

**Keywords:** consensus clustering, COVID-19, obesity, unsupervised machine learning, risk factor, risk profile discovery

## Abstract

**Introduction:**

Obesity and gender play a critical role in shaping the outcomes of COVID-19 disease. These two factors have a dynamic relationship with each other, as well as other risk factors, which hinders interpretation of how they influence severity and disease progression. This work aimed to study differences in COVID-19 disease outcomes through analysis of risk profiles stratified by gender and obesity status.

**Methods:**

This study employed an unsupervised clustering analysis, using Mexico’s national COVID-19 hospitalization dataset, which contains demographic information and health outcomes of patients hospitalized due to COVID-19. Patients were segmented into four groups by obesity and gender, with participants’ attributes and clinical outcome data described for each. Then, Consensus and PAM clustering methods were used to identify distinct risk profiles based on underlying patient characteristics. Risk profile discovery was completed on 70% of records, with the remaining 30% available for validation.

**Results:**

Data from 88,536 hospitalized patients were analyzed. Obesity, regardless of gender, was linked with higher odds of hypertension, diabetes, cardiovascular diseases, pneumonia, and Intensive Care Unit (ICU) admissions. Men tended to have higher frequencies of ICU admissions and pneumonia and higher mortality rates than women. Within each of the four analysis groups (divided based on gender and obesity status), clustering analyses identified four to five distinct risk profiles. For example, among women with obesity, there were four profiles; those with a hypertensive profile were more likely to have pneumonia, and those with a diabetic profile were most likely to be admitted to the ICU.

**Conclusion:**

Our analysis emphasizes the complex interplay between obesity, gender, and health outcomes in COVID-19 hospitalizations. The identified risk profiles highlight the need for personalized treatment strategies for COVID-19 patients and can assist in planning for patterns of deterioration in future waves of SARS-CoV-2 virus transmission. This research underscores the importance of tackling obesity as a major public health concern, given its interplay with many other health conditions, including infectious diseases such as COVID-19.

## 1 Introduction

The intersection of obesity and COVID-19 has sparked increasing concern within the global public health community. Recently, the world has observed a significant increase in obesity as a widespread public health concern, impacting millions of people from various backgrounds. Simultaneously, the emergence and rapid spread of the novel coronavirus, SARS-CoV-2, have led to a pandemic of unforeseen magnitude. As these two health crises converge, the complex relationship between obesity and COVID-19 has become a central topic for both research and discussion (1–3).

Obesity, characterized by an excessive accumulation of adipose tissue, is a major risk factor for a wide range of chronic conditions. In addition to its well-established associations with cardiovascular diseases, type 2 diabetes, and certain cancers, obesity is closely tied to several other health issues. Notably, individuals with obesity face a high risk of hypertension, the development and progression of osteoarthritis, and obstructive sleep apnea. Obesity is also connected to respiratory disorders, including asthma and decreased lung function, underscoring the broader implications for pulmonary well-being (4–10). In relation to COVID-19, evidence indicates that obesity may influence vulnerability to the virus, the severity of COVID-19 outcomes, and prolonged symptoms in individuals experiencing long-COVID, while also suggesting that individuals with obesity may be more susceptible to persistent symptoms and complications following a COVID-19 infection (11–13). This complex relationship emphasizes the urgency of addressing obesity to reduce the impact of various chronic health risks, including potential implications for long-term COVID-19 outcomes.

Furthermore, numerous other factors are thought to influence COVID-19 susceptibility and severity. Among these, gender and obesity have emerged as important areas of focus affecting COVID-19 risk (14, 15). In this study, we offer a thorough analysis of the intersection between gender, obesity status, and COVID-19 risk profiles, focusing on a population of hospitalized Mexican patients.

As COVID-19 spread worldwide, it has become increasingly evident that the virus affects people differently, with some individuals more vulnerable to severe consequences. Early research indicated a higher mortality rate in males compared to females (16–18), raising questions about the role of gender in COVID-19 susceptibility and progression. Additionally, obesity has been identified as a significant risk factor for severe COVID-19 outcomes, with multiple studies emphasizing the association between excess body weight and an increased risk of hospitalization and mortality (19–21). However, it is crucial to carry out a deeper investigation into these factors and analyze their interconnectedness within specific demographic groups.

Effectively managing the COVID-19 pandemic requires a precise understanding of the diverse risk profiles among infected individuals. Accurately outlining a subject’s risk profile is not only essential for the prompt selection of tailored treatments but also holds the potential to optimize medical resource allocation. In addition, this characterization serves as the basis for identifying and protecting the most vulnerable populations, thereby enhancing our collective ability to address the complexities of COVID-19 (22).

The primary objective of this research is to identify and comprehensively analyze risk profiles within diverse patient groups seeking medical attention, considering both obesity status and gender. Unsupervised machine learning methods are employed to reveal latent patterns that may influence health outcomes. This approach allows us to gain deeper insights into the complex interplay of factors affecting these patients, promoting a comprehensive understanding of the complicated relationships between obesity, gender, and COVID-19. Our findings may contribute to the development of targeted interventions and strategies for mitigating the impact of COVID-19, particularly in the context of obesity, in Mexico and potentially serving as a model for understanding COVID-19 risk profiles in other populations worldwide.

## 2 Materials and methods

### 2.1 Data preparation

Data were sourced from the COVID-19 Mexican Open Repository, a comprehensive database established and maintained by the Mexican government’s General Directorate of Epidemiology (23), on January 3, 2023. The Mexican government releases new data every few days, containing details on new patients and updated outcomes related to COVID-19, such as developing pneumonia, requiring admission to an ICU, and mortality due to the infection. A crucial update was made on May 9, 2023, which involved merging the newly released outcome data into our dataset to optimize patient outcomes. This update was necessary to ensure the inclusion of the latest information for a more comprehensive analysis.

This dataset contained information regarding more than 6 million subjects. These included basic demographic details such as patient identification, age, and gender. Additionally, it encompassed information on exposure history, obesity (defined as body-mass index > = 30), smoking habits, pregnancy status, and the categorization of patients into either ambulatory or hospitalized. The dataset also contained information on comorbidities such as diabetes, hypertension, cardiovascular disease, chronic obstructive pulmonary disease (COPD), asthma, immunosuppression, chronic kidney failure, and various other ailments as well as health outcomes of pneumonia, ICU admission, and death.

**Figure 1:**
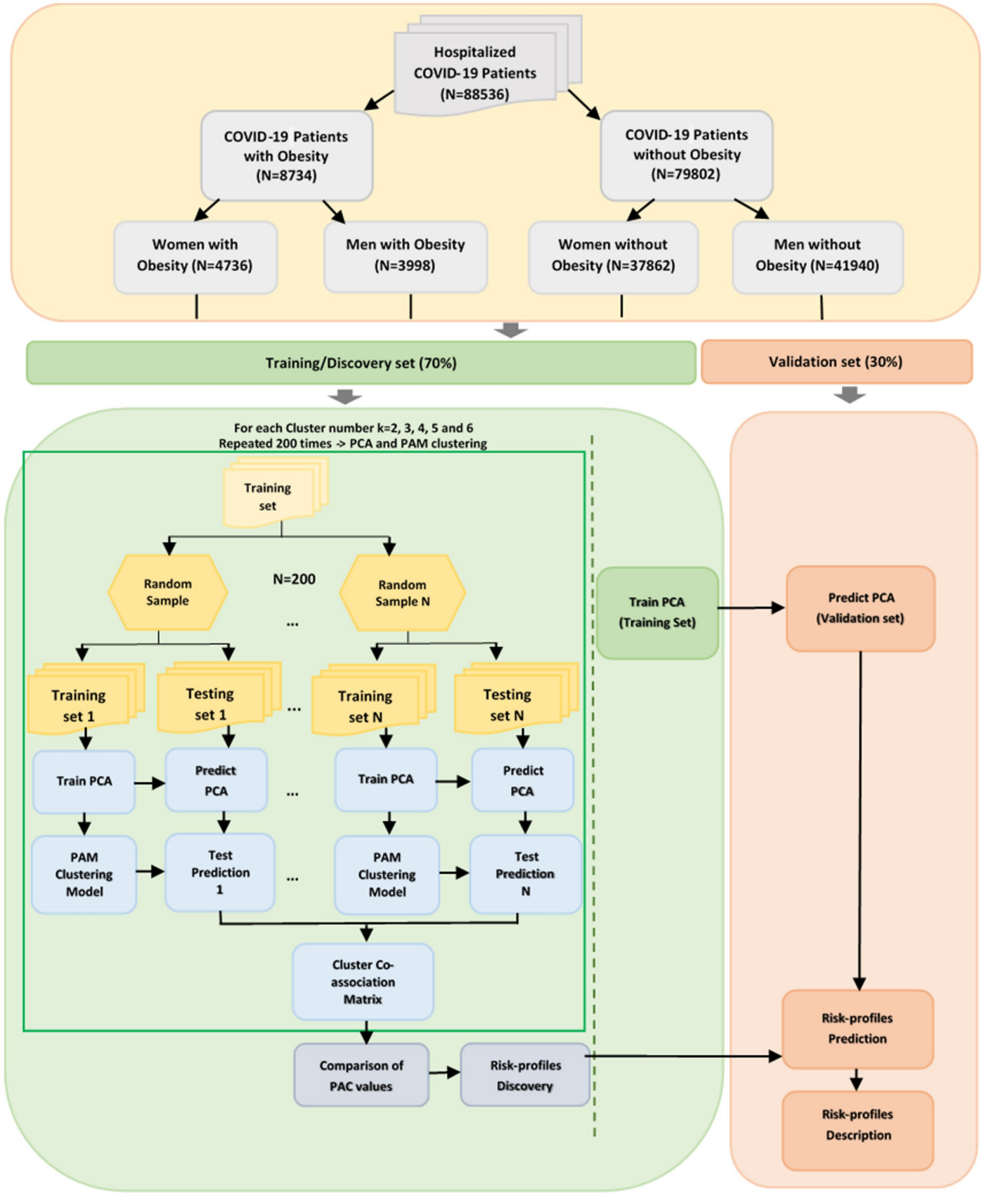
Methodology for classifying risk profiles in the Mexico COVID-19 data set.

Overall, 38 characteristics were described for each patient. Our study focused on hospitalized patients and a set of 13 risk factors crucially associated with illness severity. These factors included age, sex, obesity, smoking habits, and underlying comorbidities, as outlined in Table 1. Since our primary objective was to investigate the relationship between obesity, gender, and COVID-19, the study population was categorized into four distinct groups: women with obesity, women without obesity, men with obesity, and men without obesity as shown in Figure 1.

**Table 1:** Analysis of subject characteristics in the Mexican COVID-19 hospitalization dataset: counts (as Percentage) and mean age (standard error).

Feature	COVID subjects with obesity (*N* = 8,734)	COVID subjects without obesity (*N* = 79,802)	*p*-value	Effect size
Subjects (female ratio)	4,736 (54.22%)	37,862 (47.44%)	<0.001	OR = 1.31 (1.26–1.37)
Age	60.49 (0.18)	54.3 (0.09)	<0.001	Z = 0.28*
Pregnancy	190 (2.17%)	2,953 (3.70%)	0.37	OR **=** 0.58 (0.5–0.67)
Diabetes	4,128 (47.26%)	20,614 (25.83%)	<0.001	OR = 2.57 (2.46–2.69)**
COPD	741 (8.48%)	3,299 (4.13%)	<0.001	OR = 2.15 (1.98–2.34)**
Asthma	334 (3.82%)	1,347 (1.69%)	<0.001	OR = 2.32 (2.05–2.62)**
Immunosuppression	400 (4.58%)	2,903 (3.64%)	<0.05	OR **=** 1.27 (1.14–1.41)
Hypertension	5,262 (60.25%)	25,085 (31.43%)	<0.001	OR = 3.31 (3.16–3.46)***
Cardiovascular	1,219 (13.96%)	4,157 (5.21%)	<0.001	OR = 2.95 (2.76–3.16)**
Chronic kidney	1,137 (13.02%)	6,836 (8.57%)	<0.001	OR = 1.6 (1.49–1.71)*
Smoking	1,052 (12.04%)	4,298 (5.38%)	<0.001	OR = 2.41 (2.24–2.58)**
Other diseases	584 (6.67%)	5,070 (6.35%)	0.23	OR **=** 1.06 (0.97–1.15)
**Outcome**				
ICU	648 (7.42%)	3,949 (4.95%)	<0.001	OR = 1.54 (1.41–1.68)*
Mortality	2,925 (33.49%)	22,090 (27.68%)	<0.001	OR = 1.32 (1.26–1.38)
Pneumonia	4,764 (54.54%)	31,814 (39.87%)	<0.001	OR = 1.81 (1.73–1.89)*

Odds ratios (OR) were calculated with a 95% confidence interval, comparing COVID-19 cases in obese and non-obese individuals. Significance levels (*, **, and ***) denote effect sizes, with *, **, and *** representing small (0.2–0.5 for Z, 1.5–2 for OR), medium (0.5–0.8 for Z, 2–3 for OR), and large (>0.8 for Z, >3 for OR) effect sizes, respectively (*p*-value <0.05 indicates significant difference). The bold values represent the significance levels of the odds ratios (OR).

### 2.2 Primary statistical analysis

Hospitalized COVID-19 patients were stratified by obesity status and gender, with differences in clinical characteristics and outcomes evaluated using Cohen’s d (Z) score and Odds Ratios (OR) (24).

Moreover, the *p*-value was calculated to determine the statistical significance of the differences observed amongst groups stratified by gender and obesity status. For continuous variables, ANOVA (25) was applied, whereas the chi-square test (26) was utilized for discrete variables. A *p*-value of less than 0.05 was considered indicative of statistical significance.

### 2.3 Risk profile discovery via consensus and PAM clustering

Figure 1 presents a visual summary of the methods used to determine the risk profiles of COVID-19 patients who were hospitalized.

Each of the four patient groups was divided into a training set (70%) and a validation set (30%). The training and discovery models were established on 13 risk factors associated with COVID-19 severity. The decision to exclude outcome variables during the model-building phase was made to ensure the independence of the discovery process and to avoid introducing potential biases associated with these outcomes.

Every feature was given a normalized value ranging from 0 to 1. Specifically, the age variable was normalized across its minimum and maximum values (27). In terms of gender classification, males were coded as one, and females were designated a value of zero. For the other categorical risk factors, a value of 1 was allocated to signify the presence of the risk factor, while a value of 0 indicated its absence. Patients who had missing values for any of the 13 selected (Table 1) were excluded from the analysis.

The findings then were applied to the remaining 30% to determine how well our identified risk profiles fit them. Key to our approach was the use of techniques that clarified complex patient data into legible patterns such as the Principal Components Analysis (PCA) to reduce dimensionality, choosing PCA feature vectors that captured over 80% of the total variance (28), and grouped similar patients using the Partitioning Around Medoids (PAM) clustering method (29).

Consensus clustering and the Cluster Co-Association Matrix (CCAM) (30, 31) were utilized to assess the robustness and reliability of the clustering approach. Consensus clustering is a method that repeatedly and randomly applies a specific clustering technique to assess the reliability of the clusters it identifies, especially in the face of minor variations in the data. This repetition strengthens the clustering results, reducing sensitivity to random variations.

To ensure our clusters were robust and dependable, this process was repeated 200 times with varying cluster sizes (ranging from 2 to 6). The consistency of these clusters was evaluated using the cluster co-association matrix (CCAM), a tool that records how often two subjects end up in the same cluster. Stable groupings yield distinct patterns in the CCAM, while less reliable groupings result in blurred patterns. We quantified the clarity of these patterns using the proportion of ambiguous clustering (PAC) metric. Low PAC values suggest that the clusters are consistent and reliable, irrespective of minor changes in the data. As a result, the clustering models were trained with the optimal number of clusters determined by the most reliable grouping for four distinct patient sets within the discovery sets. The risk profiles were identified using the “Evacluster” R package (32, 33).

### 2.4 Analysis of the discovered risk-profiles

After clustering tools had identified clusters of patients within each of the four discovery patient groups, the risk profiles of the validation sets were determined. This involved:

1. Adjusting for the patients’ age to ensure comparability across different age groups.
2. Estimating the dominant health patterns or factors for each patient.
3. Assigning each patient in the validation set to a specific risk profile based on the aforementioned analysis.

Through this methodology, each patient in our validation sets was assigned a distinct label, representing their specific risk profile. After describing each risk profile, the prevalence of adverse outcomes related to each identified risk profile was analyzed. Specifically, three adverse events: pneumonia diagnosis, intensive care unit (ICU) admission, and patient death, were examined within the dataset (Table 1).

## 3 Results

### 3.1 Analysis of the Mexican COVID-19 hospitalization dataset

Our dataset comprised 88,536 patients from the Mexican COVID-19 Hospitalization Dataset who were admitted until January 3, 2023. From this cohort, 8,734 patients were categorized with obesity, while 79,802 were without obesity. Distinct differences emerged when analyzing subject characteristics and outcomes between these two groups as shown in Table 1. The odds ratios (OR) demonstrated hypertension (OR = 3.31; 95% CI: 3.16–3.46, *p* < 0.001), diabetes (OR = 2.57; 95% CI: 2.46–2.69, *p* < 0.001), and cardiovascular diseases (OR = 2.95; 95% CI: 2.76–3.16, *p* < 0.001) were significantly more frequent among people with obesity than those without obesity. Conversely, no statistically significant differences were observed in the frequency of immunosuppression or other analyzed diseases between the two groups. Specifically, the prevalence of most comorbidities among hospitalized patients with obesity was at least twice as high compared to those without obesity, with the exceptions being “other diseases” and “immunosuppression” (Supplementary Figure S1). The analysis of health outcomes showed obese patients had a remarkably higher likelihood of contracting pneumonia (OR = 1.81; 95% CI: 1.73–1.89, *p* < 0.001) and necessitating ICU admission (OR = 1.54; 95% CI: 1.41–1.68, *p* < 0.001).

### 3.2 Gender-based stratification

When studying the differences between obese men and women, significant differences emerged in risk factors between males and females such as diabetes, asthma, immunosuppression, hypertension, and cardiovascular. However, COPD, chronic kidney disease, other diseases, and age did not show these differences (Supplementary Table S1). Besides, in the non-obese cohort of patients, only ‘immunosuppression’ did not exhibit a significant gender difference (Supplementary Table S2). For outcomes such as ICU admissions, pneumonia incidence, and mortality rates, men—whether obese or not—consistently showed higher figures than women.

Furthermore, comparing across obesity status indicated all comorbidities, and outcomes, such as developing pneumonia, requiring admission to an ICU, and mortality rate for both genders were significantly more pronounced in the obese cohort. Another noteworthy observation was that both male and female patients with obesity were generally older than their non-obese counterparts. Detailed comparisons are provided in Supplementary Tables S1–S4 and Supplementary Figures S1, S2.

### 3.3 Clustering analysis results

#### 3.3.1 Validation and robustness of clusters

In our study, consensus clustering alongside the PAM clustering model was employed to identify the risk profiles of hospitalized COVID-19 patients.

Supplementary Figure S3 provides a visual representation of the optimal partitioning and PAC analysis for hypothesized 2–6 different risk profiles across all groups. The analysis of CCAM partitioning revealed that the most effective partitions exhibited distinct, well-defined patterns. Conversely, the less optimal groupings were characterized by blurred and indistinct patterns. The effectiveness of partitioning varied due to the different levels of key risk factors in each group. Specifically, how many patients in a group had a major risk factor significantly influenced the patterns seen in the CCAM partitioning. Notably, the lowest PAC value determined that the optimal partition for both women and men with obesity, as well as men without obesity, contained 4 risk profiles. In contrast, the optimal partition for women without obesity was identified to have 5 risk profiles.

Following the determination of these optimal partitions, the established risk profiles were then applied to predict the categories of the remaining 30% of patients in each group, utilizing the trained clustering models.

#### 3.3.2 Risk profiles analysis

**Figure 2:**
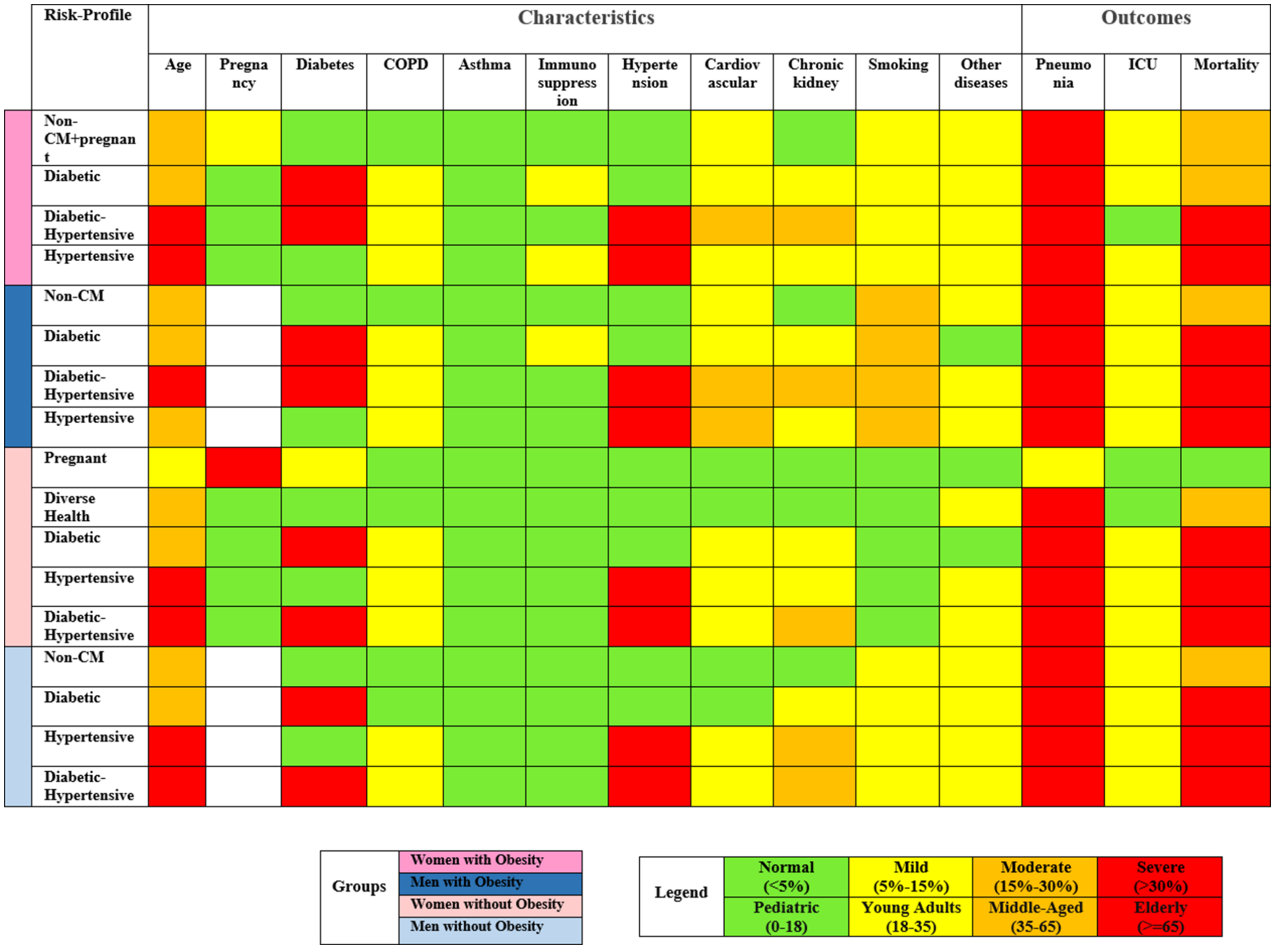
Visualization of risk factor severity in discovered risk profiles on validation sets, highlighting key characteristics within each cluster.

In obese women, four distinct profiles were discovered (Figure 2). The Non-Cardio/pregnant profile contained women without cardiometabolic diseases like diabetes or hypertension and notably had the highest proportion of pregnant women. The diabetic risk profile included only women with diabetes, while the hypertensive comprised women with hypertension. The diabetic-hypertensive profile was characterized by women with both diabetes and hypertension, displaying the highest incidence of cardiovascular diseases and chronic kidney conditions. This group also included the oldest patients (Supplementary Figure S4; Supplementary Table S5).

The risk profile among obese men closely mirrored those of obese women, divided into groups based on the presence of non-cardiometabolic conditions, diabetes, hypertension, or both diabetes and hypertension. Significantly, among these risk profiles, it is noteworthy that the prevalence of smoking was higher in men compared to women. Men without obesity also exhibited similar risk profiles to their obese counterparts (Supplementary Figures S4, S5).

Women without obesity clustered into five risk profiles. This included a group of pregnant women without significant health complications, primarily hospitalized for preventive measures. The other profiles resembled those in other cohorts, with groups for diabetes, hypertension, and both conditions and a group with a wide range of health issues (Supplementary Figure S5; Supplementary Table S7).

### 3.4 Epidemiological analysis results

#### 3.4.1 Clinical severity, ICU admissions, and mortality analysis

In Supplementary Figure S6, the clinical outcomes of hospitalized COVID-19 patients were delineated, and categorized by ICU admissions, pneumonia incidence, and mortality rates, stratified across gender and obesity status. The risk profiles were methodically arranged based on ascending mortality rates, distinguishing high-risk groups (mortality above 30%) from low-risk ones.

Among obese individuals, those in the Hypertensive risk profile experienced the highest mortality, with rates at 37.85% for women and 38.97% for men (Table 2). In contrast, non-obese patients exhibited the highest mortality within the Diabetic-Hypertensive profile, at 36.14% for women and a striking 44.24% for men. Obese women in both Hypertensive and Diabetic-Hypertensive profiles showed high mortality rates compared to their non-obese counterparts, despite being younger on average. Among men, non-obese individuals also exhibited higher mortality rates and average age compared to their obese counterparts. Overall, mortality was consistently higher in men than in women.

**Table 2:** Contribution of identified risk profiles on validation sets to clinical severity, ICU admissions, and mortality rates, with bold values, indicating the highest outcome in each patient group.

	Risk profiles	% of patients	% of people who were admitted to ICU	% of people who died	% of people who had Pneumonia
Women with obesity (*N* = 1,421) 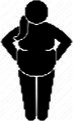	Non-CM + pregnant	26.6%	7.14%	21.96%	45.50%
	Diabetic	11.26%	**10%**	30%	52.50%
	Diabetic-hypertensive	**37.23%**	3.97%	36.48%	53.31%
	Hypertensive	24.91%	5.65%	**37.85%**	**57.34%**
Men with obesity (*N* = 1,200) 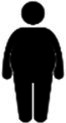	Non-cardiometabolic	31.58%	9.23%	29.29%	57.78%
	Diabetic	10%	**12.5%**	36.67%	**62.5%**
	Diabetic-hypertensive	**35.75%**	6.99%	38.69%	62.47%
	Hypertensive	22.67%	8.82%	**38.97%**	56.98%
Women without obesity (*N* = 11,359) 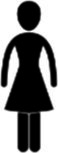	Pregnant	7.73%	2.28%	0.34%	14.12%
	Diverse health	**55.06%**	3.85%	16.74%	30.24%
	Diabetic	7.31%	5.54%	30.84%	45.78%
	Hypertensive	13.49%	5.09%	33.68%	46.61%
	Diabetic-hypertensive	16.42%	**5.65%**	**36.14%**	**49.22%**
Men without obesity (*N* = 12,583) 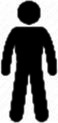	Non-cardiometabolic	**59.32%**	5.26%	25.68%	37.62%
	Diabetic	9.09%	7.51%	41.52%	**52.81%**
	Hypertensive	14.2%	**7.61%**	41.75%	52.55%
	Diabetic-hypertensive	17.39%	6.41%	**44.24%**	52.24%

Furthermore, our analysis revealed significant trends in other clinical outcomes. Specifically, obese diabetic patients, both men and women, showed a heightened need for ICU admission with 12.5 and 10%, surpassing other risk profiles. Those in the diabetic hypertensive category had the least ICU admissions. Also, non-obese patients, regardless of gender, generally required less ICU intervention compared to obese patients as shown in Table 2.

Besides, our study identified clear trends in pneumonia incidence. Patients with obesity, irrespective of gender, had a higher predisposition to pneumonia following COVID-19 infection. Additionally, men, whether obese or not, displayed a greater tendency to develop pneumonia than women (Table 2).

The analysis of risk profiles in hospitalized COVID-19 patients, stratified by gender and obesity status, revealed complicated interactions among diverse factors, leading to variations in selected comorbidities and outcomes across the identified risk profiles within the four patient groups. Notably, factors such as asthma, immunosuppression, smoking, and other diseases did not play a significant role in distinguishing these risk profiles. However, an exception was observed in women without obesity, where only the prevalence of asthma remained relatively consistent across different risk profiles (see Supplementary Tables S5–S8).

In-depth comparisons of effect sizes enhance understanding of prominent risk factors critical for patient categorization within each risk profile. These analyses indicated that, apart from primary risk factors like diabetes and hypertension, other elements significantly contribute to delineating high-risk from low-risk profiles. Notably, cardiovascular issues, chronic kidney diseases, and COPD (Chronic Obstructive Pulmonary Disease) are paramount in distinguishing low-risk profiles from hypertensive and diabetic-hypertensive profiles in all patient groups. For women without obesity, the presence of additional diseases is also instrumental in differentiating between risk profiles (see Supplementary Tables S9–S12).

#### 3.4.2 Outcome-specific risk ratios

**Figure 3:**
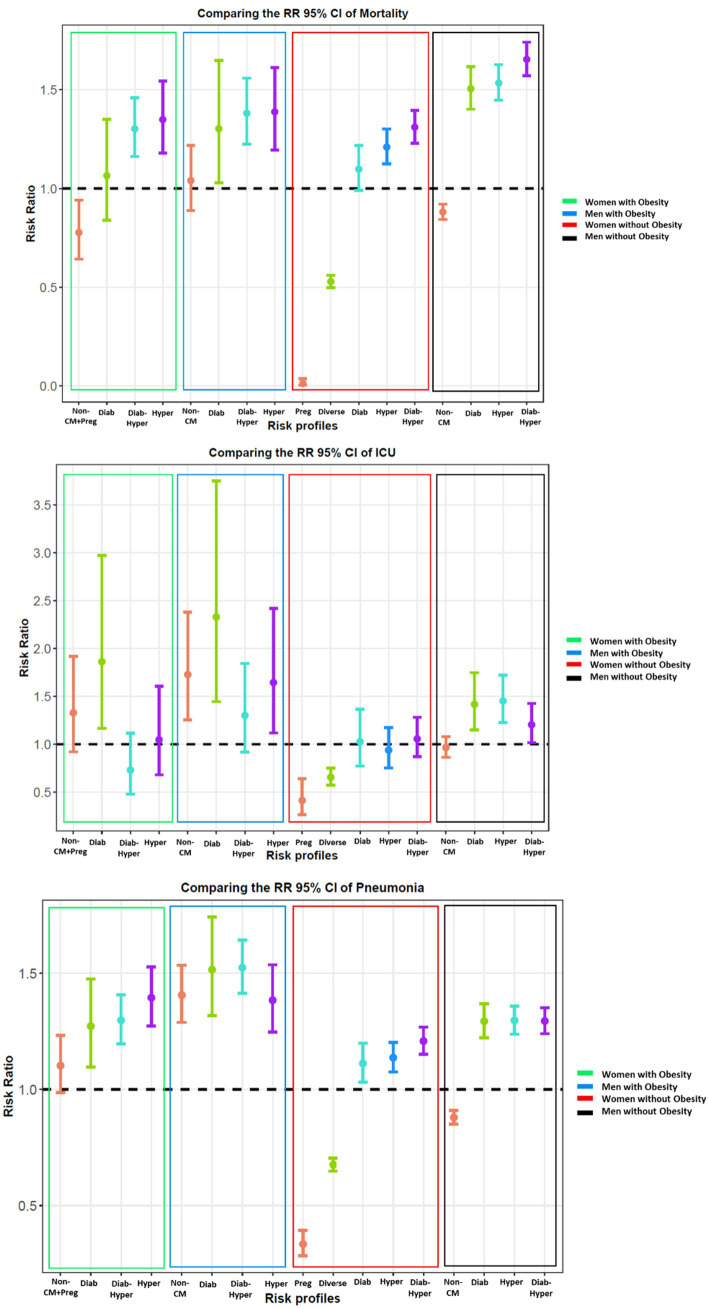
Comparison of risk ratios (95% confidence intervals) of the outcomes (the rate of mortality, ICU, and pneumonia) among four categories of validation sets: women with and without obesity, men with and without obesity, for discovered risk profiles.

In our comprehensive analysis, the varied risk ratios of all outcomes also were explored, with a 95% confidence interval, across different risk profiles for hospitalized COVID-19 patients, within the four patient groups (Figure 3). The high-risk profiles among non-obese men showed significantly higher risk rates of mortality, especially in those with both diabetes and hypertension. Conversely, women without obesity in high-risk profiles exhibited the lowest mortality risk ratios.

In addition, distinct patterns concerning ICU admissions and pneumonia were observed (Figure 3). Notably, obese patients, regardless of gender, faced higher risk ratios for ICU admission and pneumonia across all risk profiles. Even low-risk profiles within obese groups, such as Non-Cardiometabolic and Non-Cardiometabolic/Pregnant, showed higher needs for ICU admission than those in higher-risk categories like Hypertensive and Diabetic-Hypertensive. This trend was particularly pronounced given the younger age of these low-risk individuals. Conversely, low-risk profiles among non-obese individuals that constituted the youngest demographic displayed no significant risk ratios. Moreover, our data consistently showed that outcomes were more severe for men, irrespective of obesity status, compared to women.

#### 3.4.3 Regional prevalence patterns

**Figure 4:**
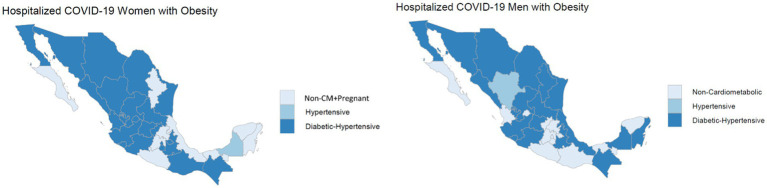
Geographical variation in risk profiles: highlighting the state with the highest frequency of risk profiles.

Figure 4 illustrates the geographical distribution and prevalence of risk profiles among obese patients, revealing notable regional variations in the most frequent risk profiles between men and women. Specifically, in certain southern states of Mexico, distinct risk profiles emerge with varying frequencies between men and women in these regions, including diabetic-hypertensive, non-cardiometabolic conditions, or Non-CM + Pregnant. In contrast, the northern states exhibit a more consistent pattern, with the most frequent risk profiles such as diabetic-hypertensive that is nearly identical between different genders. The distribution of risk profiles and their corresponding geographical locations within the validation set, focusing on hospitalized COVID-19 patients with obesity, is provided in Supplementary Tables S17, S18 for a comprehensive understanding.

#### 3.4.4 Centenarian subgroup analysis

In our study, a small cohort of centenarians, individuals aged 100 years or older, were observed among both the obese and non-obese patient groups. A remarkable finding occurred: all centenarian patients with obesity succumbed to COVID-19, illustrating a complete mortality rate in this subgroup. In contrast, among the non-obese centenarians, there were instances of survival, with a calculated survival rate of 0.47. Although the difference in survival rates between the obese (0%) and non-obese (47%) centenarians was not statistically significant (*p* = 0.54), it is important to note the small sample size as a limitation of the analysis.

## 4 Discussion

### 4.1 The impact of obesity on COVID-19 outcomes

Our study provides an extensive analysis of the complicated relationship between obesity and COVID-19 outcomes, highlighting the substantial influence of obesity on the disease’s progression. Notably, the odds ratios demonstrate that obesity is associated with an increased risk for conditions like hypertension, diabetes, and cardiovascular diseases, which are known contributors to COVID-19 complications (Table 1). These conditions are not only prevalent comorbidities but also play a significant role in exacerbating COVID-19 complications.

Moreover, the observation that obese patients have a higher tendency for ICU admission and are more prone to pneumonia is particularly striking. Our previous analysis, clustering all patients without stratification, demonstrated that obesity was a factor for some of the high-risk profiles (34). After the stratification of patients, we were able to identify obesity as a pivotal determinant in the escalation of COVID-19 severity (pneumonia and ICU admissions). This finding aligns with existing research, indicating that obesity exacerbates the prognosis of infectious diseases, highlighting its role as an exacerbating factor due to associated comorbidities and impaired immune responses (35, 36).

### 4.2 Gender disparities in COVID-19 hospitalizations

Our analysis reveals notable gender differences. Men, regardless of obesity status, consistently exhibited worse outcomes, including higher ICU admissions, developing pneumonia, and mortality rates (Supplementary Figures S1, S2). This observation is in harmony with studies indicating a higher susceptibility of men to severe COVID-19 outcomes (37, 38). Various hypotheses have been proposed to explain this trend. Biological differences between genders, such as variations in immune system responses, might play a role (39). For instance, some studies indicate that sex hormones and genetic factors could influence the immune response, potentially making men more susceptible to severe infections (40). Lifestyle factors, including higher rates of smoking and alcohol consumption often observed in men, might contribute to this difference (41, 42). Underlying health conditions prevalent among men, such as cardiovascular diseases, could exacerbate COVID-19 outcomes (43). The observation that non-obese men had higher mortality rates compared to obese ones is intriguing and suggests the complexity of risk factors influencing COVID-19 outcomes.

#### 4.2.1 Justification for effect modifiers

Our choice of gender and obesity as effect modifiers stems from both the specific focus of our institute on obesity research and the compelling evidence supporting these factors as critical determinants of COVID-19 outcomes. The extensive literature linking obesity to increased severity of infectious diseases, coupled with observed gender disparities in susceptibility and outcomes, justified our decision to explore the interplay of these factors in our clustering analysis. By considering these effect modifiers, our study provides a more nuanced understanding of the various risk profiles within the COVID-19 population.

### 4.3 Risk profiles: a nuanced understanding of COVID-19 risks

The utilization of clustering methods in our study has provided a nuanced understanding of COVID-19 risk profiles, emphasizing the complex interplay of factors influencing disease outcomes. Our findings reveal significant variations in risk profiles based on gender and obesity status, highlighting the complex landscape of risk determinants. Notably, distinct risk profiles, especially among non-obese women, underscore the inherent heterogeneity among COVID-19 patients, necessitating personalized medical strategies tailored to each patient’s unique combination of risk factors. This approach enhances patient care by enabling more focused treatments aligned with specific risks, contributing to a healthcare system prioritizing individual patient needs.

Moreover, our analysis found that certain factors, including asthma, immunosuppression, smoking, and other comorbidities, did not significantly alter COVID-19 risk profiles (Figure 2). This finding indicates the need for a thorough investigation into the primary drivers of severe COVID-19 outcomes, prompting a re-evaluation of the importance attributed to various risk factors in clinical decision-making.

Our comprehensive analysis across risk profiles provided valuable insights into varied outcomes for hospitalized COVID-19 patients, revealing noteworthy patterns within the four patient groups and emphasizing the complexity of factors influencing outcomes (Figure 3).

Significantly higher mortality risk among non-obese men in high-risk profiles, particularly with diabetes and hypertension, highlights a concerning gender disparity. Conversely, women without obesity in high-risk profiles exhibited the lowest mortality risk, emphasizing the need for personalized and gender-sensitive healthcare strategies.

Distinct patterns in ICU admissions and pneumonia revealed significant implications for patient care. Obese patients, irrespective of gender, faced higher risk ratios across all risk profiles. Notably, even low-risk profiles in obese groups showed a higher incidence of ICU admissions compared to higher-risk profiles in other categories, particularly pronounced in the younger age group. Specifically, diabetic risk profiles among obese patients exhibited the highest need for admission to the ICU, emphasizing the critical importance of tailored interventions for this subgroup.

The integration of consensus clustering and the PAM model has enabled a comprehensive study of the complex nature of COVID-19 risk factors, facilitating a refined approach to patient categorization. This methodology has demonstrated notable strength in predicting patients’ risk profiles, considering various combinations of risk factors across diverse patient groups.

### 4.4 Geographical variations in risk profiles

The geographical variation in risk profiles among obese patients, shown in Figure 4, suggests the influence of regional factors, possibly including healthcare accessibility, lifestyle, and demographic differences. The significant regional differences underline the importance of localized healthcare strategies and resource allocation.

Nevertheless, the nature of regional disparities necessitates careful interpretation, considering factors such as demographics and healthcare infrastructure. The findings emphasize the ongoing importance of care and a comprehensive public health strategy, incorporating preventive measures alongside vaccination efforts, to effectively navigate the evolving landscape of the pandemic and sustain positive outcomes.

In addition, identifying and understanding the risk profiles for COVID-19 in different local areas and cities is critical to developing targeted and effective pandemic management strategies. Risk profiling allows for a tailored approach that considers the unique characteristics of each locality, promoting more efficient resource allocation and improving overall readiness.

### 4.5 The centenarian cohort: a glimpse into extreme age and COVID-19

The observation that all centenarian patients with obesity succumbed to COVID-19, while some non-obese centenarians survived, is striking and provides valuable insights into the interactions between extreme age, obesity, and COVID-19 survivability (31). This prompts questions about the physiological and immune system changes in centenarians, especially in the presence of obesity. However, it is crucial to acknowledge the inherent limitations of the same sample size of this cohort. Despite these limitations, our study serves as a preliminary exploration into a relatively uncharted area of research involving centenarians and their susceptibility to COVID-19.

### 4.6 Implications for healthcare planning and future preparedness

Our research focuses on identifying and characterizing specific risk profiles among individuals impacted by COVID-19. Beyond the immediate implications for treatment selection, our findings offer insights for planning and preparation, a proactive approach to preventing hospitals from being overwhelmed in the face of potential future waves of severe COVID-19. The research contributes to our understanding of risk profiles and gives practical insights for strengthening the healthcare system against future challenges.

### 4.7 Limitations and future works

Our study, while offering valuable insights, presents limitations. One significant concern is that all subjects are hospitalized patients, which may not accurately reflect the entire spectrum of individuals affected by COVID-19. To address this, future research could expand the scope to include data from outpatient departments, providing a more comprehensive view of the impact of obesity on COVID-19 outcomes across different patient populations. Longitudinal follow-up studies would also be beneficial to understand the long-term effects of obesity on COVID-19 prognosis.

Furthermore, our study was based on a Mexican cohort primarily composed of individuals seeking medical care or those who were hospitalized. This aspect potentially limits the generalizability of our findings to the broader population. Consequently, validating these findings in a separate, more diverse cohort is essential for broader applicability.

Another limitation lies in our cluster-based analytical approach. While this method successfully identified significant risk profiles, it may have overlooked other relevant conditions. In addition, the number of variables is limited. The patient record did not include body-mass index (BMI) as a numerical variable, nor other metabolic features, which may be critical to understanding the effect of obesity on COVID-19 infection and aid our clustering algorithm. Lastly, it is important to note that outcomes, treatment protocols, and the understanding of SARS-CoV-2 subtypes have evolved during the COVID-19 pandemic. The variability in hospital saturation levels and the introduction of new treatments during the pandemic may have influenced our findings. Therefore, the associations identified in our study may be most applicable to the specific population and period we examined.

## 5 Conclusion

In summary, our study illuminates the complex interactions between factors influencing COVID-19 outcomes, with a particular emphasis on the role of obesity and gender differences. The findings highlight the importance of recognizing obesity not merely as an isolated risk factor but as a pivotal element that interacts with other health conditions, exacerbating COVID-19 severity. This understanding is crucial for devising personalized treatment strategies, improving patient care, and improving public health. Additionally, the application of consensus clustering and the PAM model in our study has paved the way for a more detailed and nuanced understanding of COVID-19 risk factors, enabling a more precise categorization of patients and informing more effective healthcare strategies.

## Data availability statement

Publicly available datasets were analyzed in this study. This data can be found at: Datos Abiertos Dirección General de Epidemiología https://datosabiertos.salud.gob.mx/gobmx/salud/datos_abiertos/datos_abiertos_covid19.zip.

## Author contributions

FN: Conceptualization, Data curation, Methodology, Software, Visualization, Writing – original draft, Writing – review & editing. JT-P: Conceptualization, Supervision, Writing – review & editing. EM-L: Conceptualization, Funding acquisition, Methodology, Supervision, Validation, Writing – original draft, Writing – review & editing.
