# Supplementary material for: Exploring the intersection of obesity and gender in COVID-19 outcomes in hospitalized Mexican patients: a comparative analysis of risk profiles using unsupervised machine learning
Source: Front Public Health. 2024 Apr 18;12:1337432. doi: 10.3389/fpubh.2024.1337432 (PMC11063238; doi:10.3389/fpubh.2024.1337432)
Supplement: Supplementary file 1 [file Data_Sheet_1.PDF]

Supplementary Material

Exploring the Intersection of Obesity and Gender in COVID-19 Outcomes in hospitalized Mexican patients: A Comparative Analysis of Risk Profiles Using Unsupervised Machine Learning

Fahimeh Nezhadmoghadam, José Gerardo Tamez-Peña, Emmanuel Martinez-Ledesma*

*** Correspondence:** Emmanuel Martinez-Ledesma, juanemmanuel@tec.mx

1 Results

1.1 Gender-Based Stratification

Supplementary Table 1. Analysis of Subject Characteristics in the Mexican COVID-19 Hospitalization Dataset Stratified by Gender and Obesity Status: Counts (as Percentage) and Mean Age (Standard Error). Odds ratios (OR) were calculated with a 95% confidence interval, comparing COVID-19 cases in obese and non-obese individuals. Significance levels (*, **, and ***) denote effect sizes, with *, **, and *** representing small (0.2-0.5 for Z, 1.5-2 for OR), medium (0.5-0.8 for Z, 2-3 for OR), and large (>0.8 for Z, >3 for OR) effect sizes, respectively. (P- Value < 0.05 indicates significant difference)

Feature	COVID Women with Obesity (N=4736)	COVID Men with Obesity (N=3998)	P-value	Effect Size
Age	60.5 (0.26)	60.47 (0.27)	0.94	Z=0.002
Pregnancy	190 (4.01%)	0	<0.001	
Diabetes	2290 (48.35%)	1838 (45.97%)	<0.05	OR=1.1 (1.01- 1.2)
COPD	409 (8.30%)	332 (8.64%)	0.61	OR=1.04 (0.9- 1.21)
Asthma	230 (4.86%)	104 (2.60%)	<0.001	**OR=1.91 (1.51- 2.42)***
Immunosuppression	239 (5.05%)	161 (4.03%)	<0.05	OR=1.27 (1.03- 1.55)
Hypertension	2910 (61.44%)	2352 (58.83%)	<0.05	OR=1.12 (1.02- 1.22)
Cardiovascular	609 (12.86%)	610 (15.26%)	<0.05	OR=0.82 (0.73- 0.93)
Chronic kidney	607 (12.82%)	530 (13.26%)	0.56	OR=0.96 (0.85- 1.09)
Smoking	351 (7.41%)	701 (17.53%)	<0.001	OR=0.38 (0.33- 0.43)
Other diseases	337 (7.12%)	247 (6.18%)	0.09	OR=1.16 (0.98- 1.38)
**Outcome**				

ICU	310 (6.55%)	338 (8.46%)	<0.001	OR=0.76 (0.65- 0.89)
Deaths	1474 (31.12%)	1451 (36.29%)	<0.001	OR=0.79 (0.73- 0.87)
Pneumonia	2448 (51.69%)	2316 (57.93%)	<0.001	OR=0.78 (0.71- 0.85)

Supplementary Table 2. Analysis of Subject Characteristics in the Mexican COVID-19 Hospitalization Dataset for Patients without Obesity, Stratified by Gender: Counts (as Percentage) and Mean Age (Standard Error). Odds ratios (OR) were calculated with a 95% confidence interval, comparing COVID-19 cases in obese and non-obese individuals. Significance levels (*, **, and ***) denote effect sizes, with *, **, and *** representing small (0.2-0.5 for Z, 1.5-2 for OR), medium (0.5-0.8 for Z, 2-3 for OR), and large (>0.8 for Z, >3 for OR) effect sizes, respectively. (P- Value < 0.05 indicates significant difference)

**Feature**	**COVID Women without Obesity (N=37862)**	**COVID Men without Obesity (N=41940)**	**P-value**	**Effect Size**
Age	52.01 (0.13)	56.38 (0.13)	<0.001	Z=-33.61
Pregnancy	1518 (4.01%)	0	<0.001	
Diabetes	9367 (24.74%)	11247 (26.82%)	<0.001	OR=0.9 (0.87- 0.93)
COPD	1463 (3.86%)	1836 (4.38%)	<0.001	OR=0.88 (0.82- 0.94)
Asthma	806 (2.13%)	541 (1.29%)	<0.001	**OR=1.66 (1.49- 1.86)***
Immunosuppression	1386 (3.66%)	1517 (3.62%)	0.76	OR=1.01 (0.94- 1.09)
Hypertension	11570 (30.56%)	13515 (32.22%)	<0.001	OR=0.93 (0.9- 0.95)
Cardiovascular	1729 (4.57%)	2428 (5.79%)	<0.001	OR=0.78 (0.73- 0.83)
Chronic kidney	2810 (7.42%)	4026 (9.60%)	<0.001	OR=0.75 (0.72- 0.79)
Smoking	981 (2.59%)	3317 (7.91%)	<0.001	OR=0.31 (0.29- 0.33)
Other diseases	2542 (6.71%)	2528 (6.03%)	<0.001	OR=1.12 (1.06- 1.19)
**Outcome**				
ICU	1622 (4.28%)	2327 (5.55%)	<0.001	OR=0.76 (0.71- 0.81)
Deaths	8555 (22.59%)	13535 (32.27%)	<0.001	OR=0.61 (0.59- 0.63)
Pneumonia	13604 (35.93%)	18210 (43.42%)	<0.001	OR=0.73 (0.71- 0.75)

Supplementary Table 3. Analysis of Subject Characteristics in the Mexican COVID-19 Hospitalization Dataset for Women with/without Obesity. (P- Value < 0.05 indicates significant difference)

**Feature**	**Hospitalized COVID-19 Women**		**P-value**
	**with Obesity (N=37862)**	**without Obesity (N=41940)**	
Age	60.5 (0.26)	52.01 (0.13)	<0.001
Pregnancy	190 (4.01%)	1518 (4.01%)	<0.001
Diabetes	2290 (48.35%)	9367 (24.74%)	<0.001
COPD	409 (8.30%)	1463 (3.86%)	<0.001
Asthma	230 (4.86%)	806 (2.13%)	<0.001
Immunosuppression	239 (5.05%)	1386 (3.66%)	<0.001
Hypertension	2910 (61.44%)	11570 (30.56%)	<0.001
Cardiovascular	609 (12.86%)	1729 (4.57%)	<0.001
Chronic kidney	607 (12.82%)	2810 (7.42%)	<0.001
Smoking	351 (7.41%)	981 (2.59%)	<0.001
Other diseases	337 (7.12%)	2542 (6.71%)	0.31
**Outcome**			
ICU	310 (6.55%)	1622 (4.28%)	<0.001
Deaths	1474 (31.12%)	8555 (22.59%)	<0.001
Pneumonia	2448 (51.69%)	13604 (35.93%)	<0.001

Supplementary Table 4. Analysis of Subject Characteristics in the Mexican COVID-19 Hospitalization Dataset for Men with/without Obesity. (P- Value < 0.05 indicates significant difference)

Feature	Hospitalized COVID-19 Men		P-value
	with Obesity (N=37862)	without Obesity (N=41940)	
Age	60.47 (0.27)	56.38 (0.13)	<0.001
Diabetes	1838 (45.97%)	11247 (26.82%)	<0.001
COPD	332 (8.64%)	1836 (4.38%)	<0.001
Asthma	104 (2.60%)	541 (1.29%)	<0.001
Immunosuppression	161 (4.03%)	1517 (3.62%)	0.20
Hypertension	2352 (58.83%)	13515 (32.22%)	<0.001
Cardiovascular	610 (15.26%)	2428 (5.79%)	<0.001
Chronic kidney	530 (13.26%)	4026 (9.60%)	<0.001
Smoking	701 (17.53%)	3317 (7.91%)	<0.001
Other diseases	247 (6.18%)	2528 (6.03%)	0.73
**Outcome**			
ICU	338 (8.46%)	2327 (5.55%)	<0.001
Deaths	1451 (36.29%)	13535 (32.27%)	<0.001
Pneumonia	2316 (57.93%)	18210 (43.42%)	<0.001

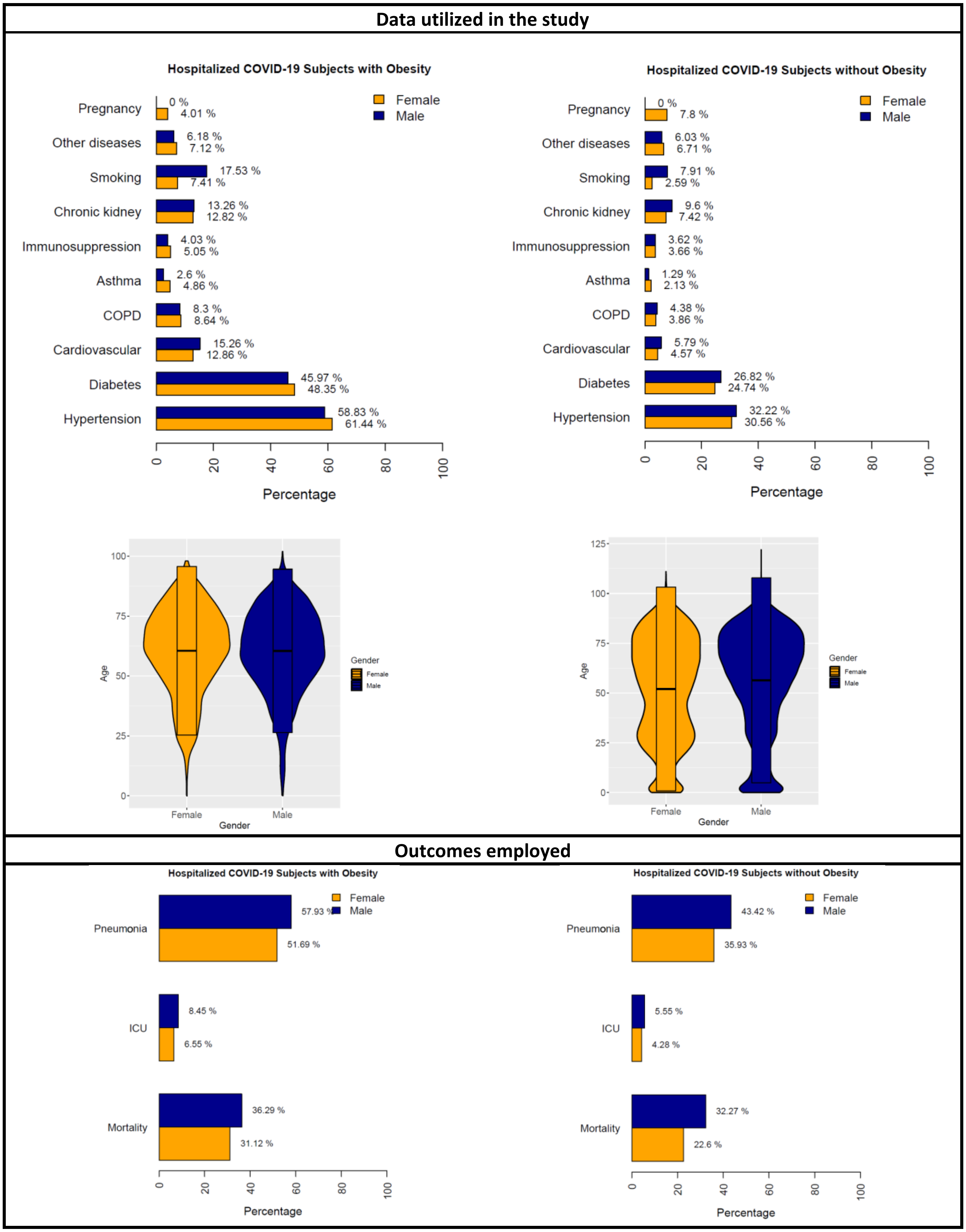

Supplementary Figure 1. Comparing Hospitalized COVID Patients: Study Data and Outcomes for Women and Men with/without Obesity.

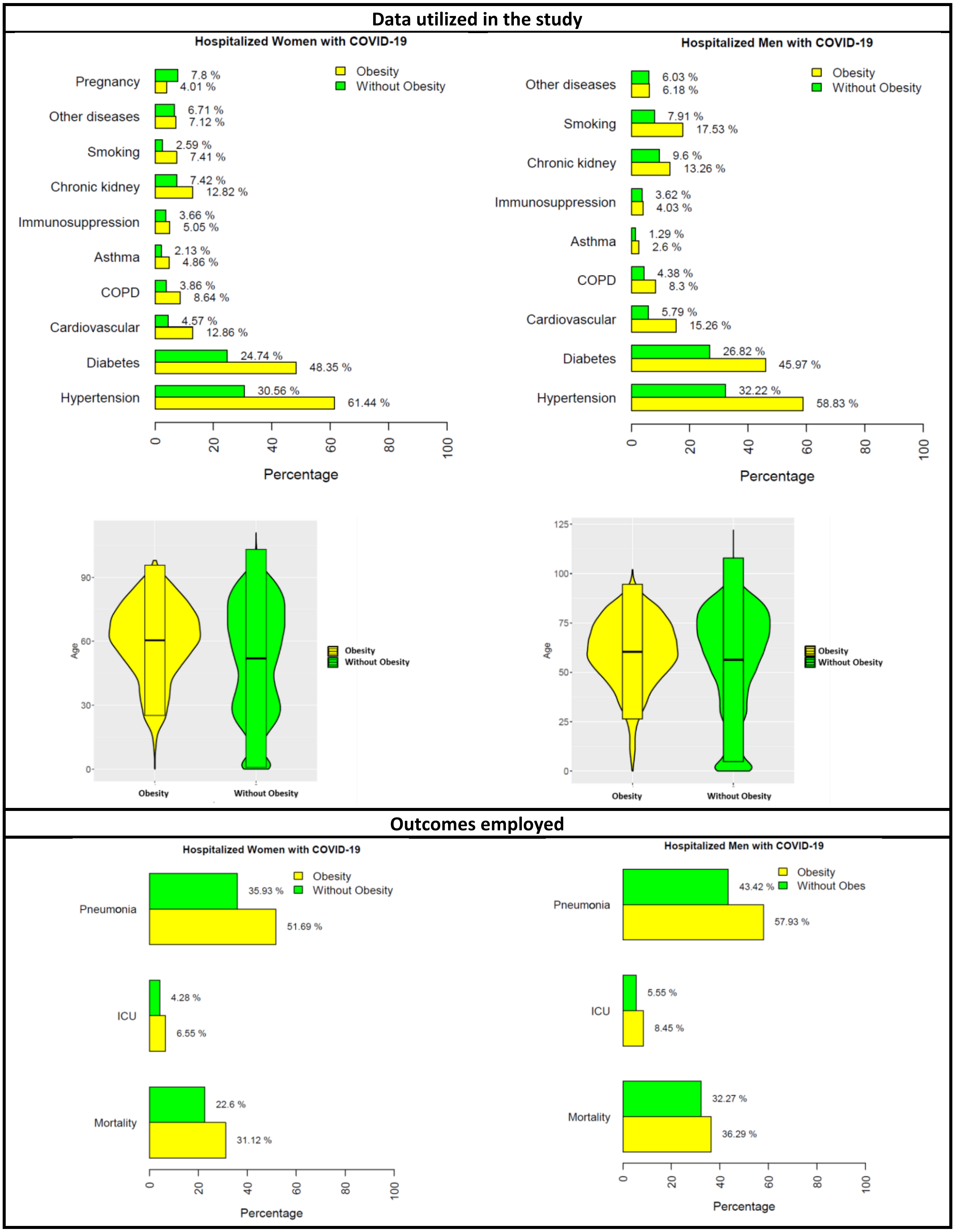

Supplementary Figure 2. COVID Hospitalized Patients: Comparing Outcomes by Gender and Obesity Status.

1.2 Clustering and Risk Profile Analysis

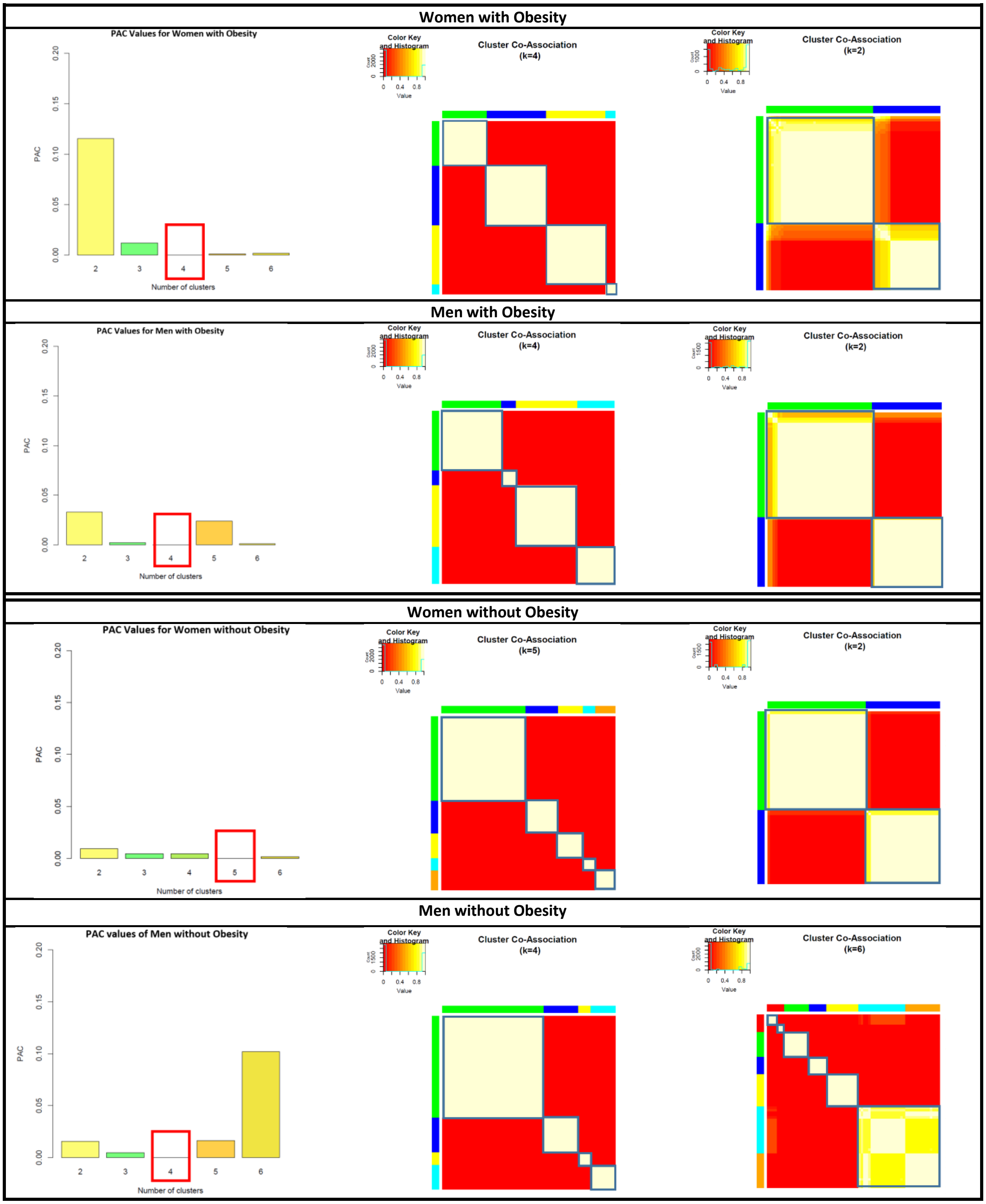

Supplementary Figure 3. Consensus Clustering Analysis of hospitalized COVID-19 patients (Women and Men with/without obesity): left) PAC Comparison, middle) Best Consensus Result, and right) Worst Results.

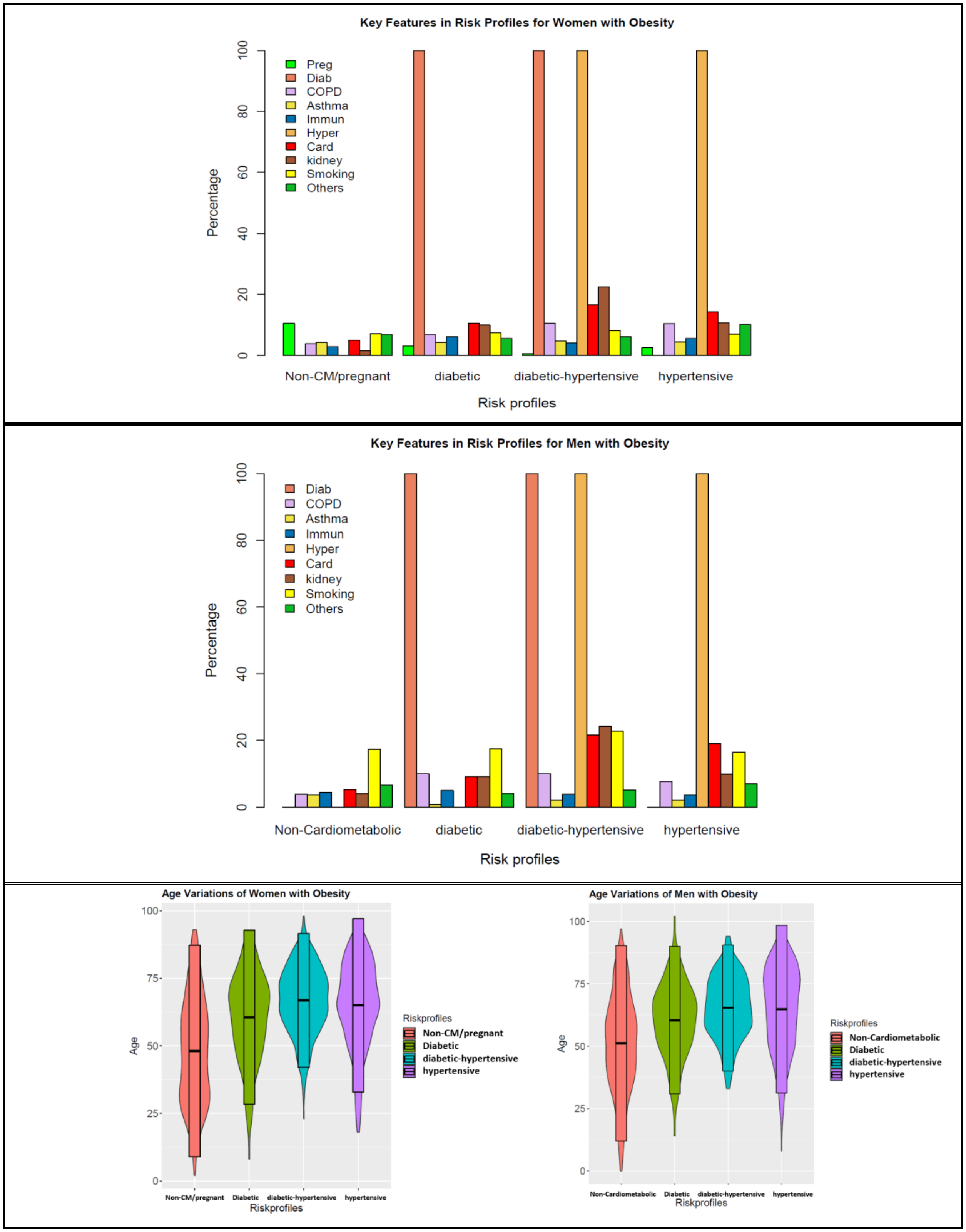

Supplementary Figure 4. Comparative Analysis of Selected Feature Distribution among Risk Profiles for Hospitalized Patients with Obesity, Stratified by Gender on Validation sets.

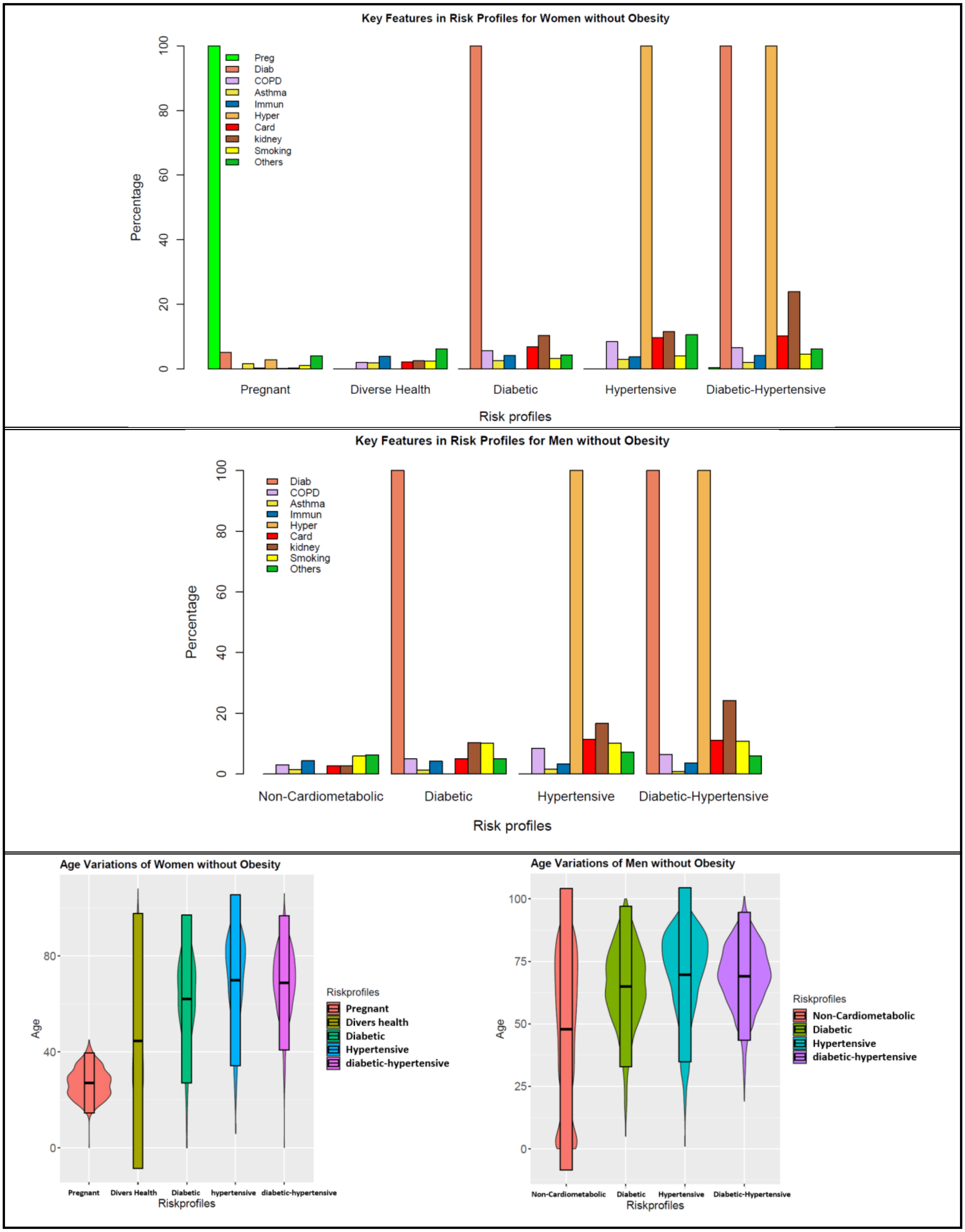

Supplementary Figure 5. Comparative Analysis of Selected Feature Distribution among Risk Profiles for Hospitalized Patients without Obesity, Stratified by Gender on Validation sets.

Covid-19 Hospitalized Patients with Obesity

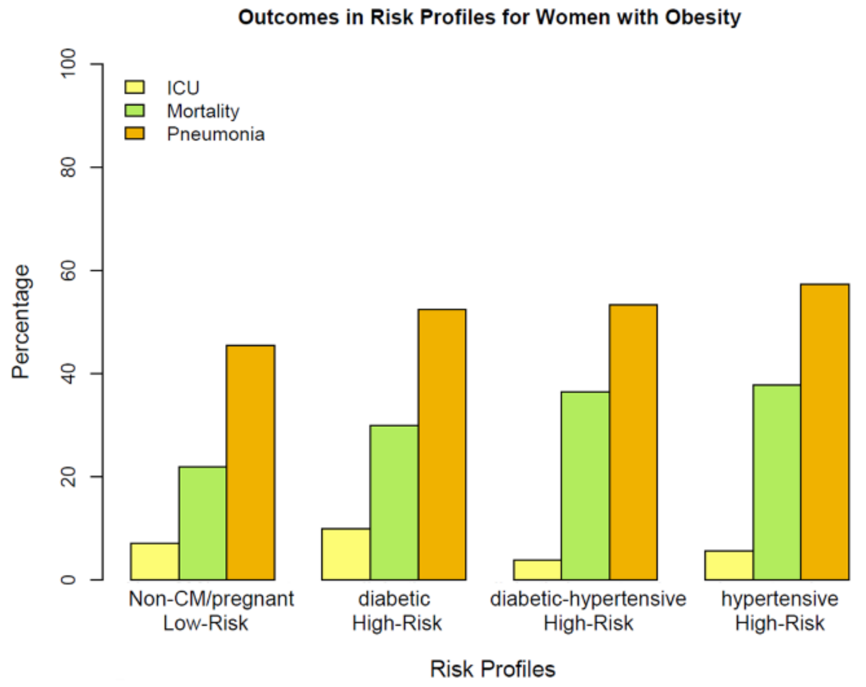

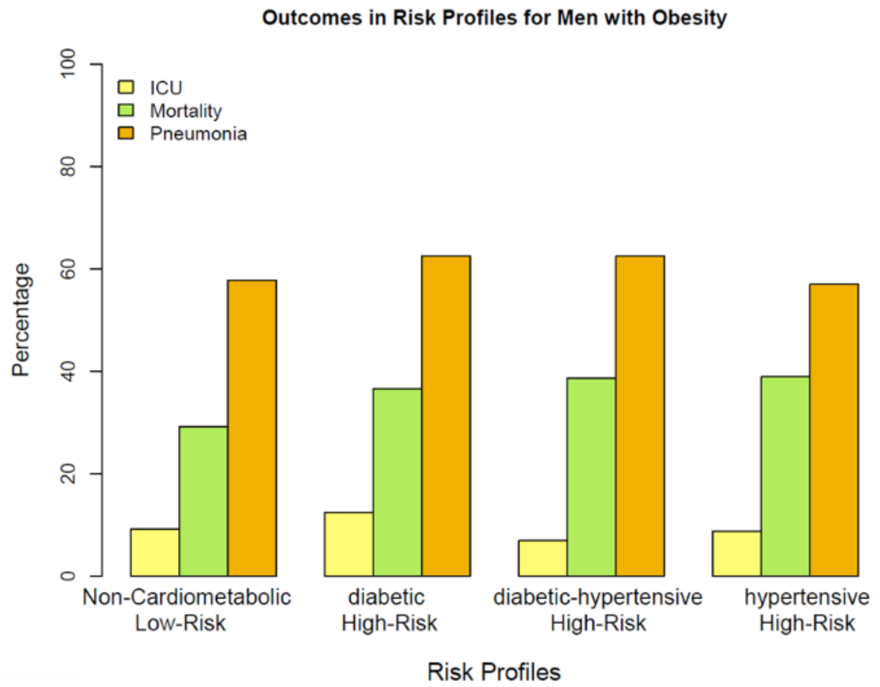

Covid-19 Hospitalized Patients without Obesity

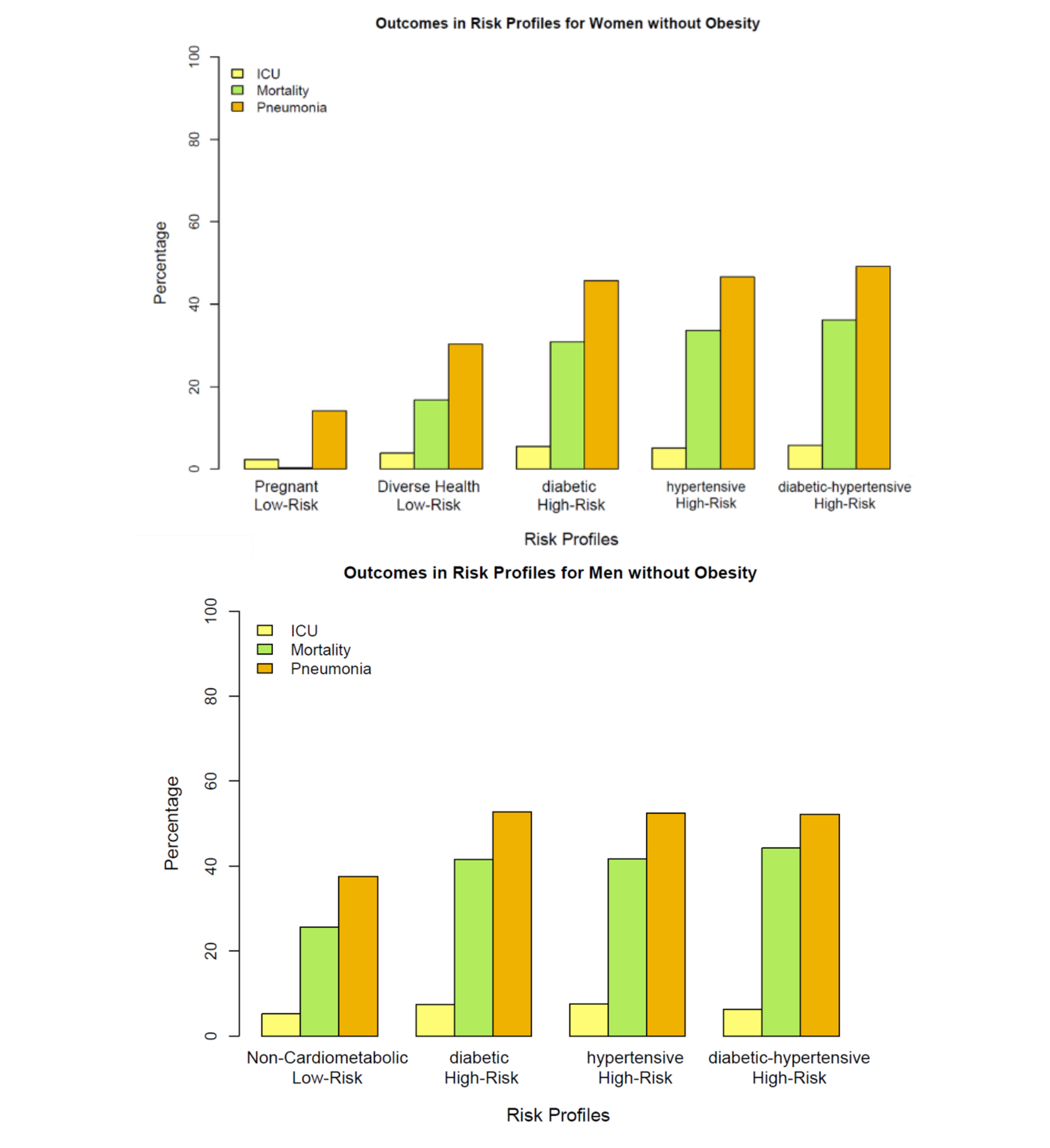

Supplementary Figure 6. Comparative Analysis of Validation Set Outcomes for Hospitalized Patients: Stratified by Gender and Obesity Status across Various Risk Profiles on Validation sets.

Supplementary Table 5. Characterizing Risk Profiles in Hospitalized COVID-19 Women with Obesity on Validation sets: Insights from Validation Set Analysis of Selected Characteristics, Age Distribution, and Statistical Significance. We calculated the prevalence (as a percentage) of individuals exhibiting specific characteristics and determined the mean age (with standard error) within each risk profile. To assess statistical significance, we employed ANOVA and chi-squared tests for continuous and discrete variables, respectively, comparing differences between risk profiles.

**Feature**	**Non-CM/pregnant (Low-Risk) (N=378)**	**Diabetic (High-Risk) (N=160)**	**Diabetic-Hypertensive (High-Risk) (N=529)**	**Hypertensive (High-Risk) (N=354)**	**p-value**
Age	48.07 (1.01)	60.56 (1.27)	66.83 (0.54)	65.05 (0.85)	**<0.05**
Pregnancy	40 (10.58%)	5 (3.12%)	3 (0.57%)	9 (2.54%)	**<0.001**
Diabetes	0	160 (100%)	529 (100%)	0	**<0.001**
COPD	15 (3.97%)	11 (6.87%)	56 (10.57%)	37 (10.45%)	**<0.05**
Asthma	16 (4.23%)	7 (4.37%)	25 (4.73%)	16 (4.52%)	0.99
Immunosuppression	11 (2.91%)	10 (6.25%)	22 (4.16%)	20 (5.65%)	0.2
Hypertension	0	0	529 (100%)	354 (100%)	**<0.001**
Cardiovascular	19 (5.03%)	17 (10.62%)	88 (16.63%)	51 (14.41%)	**<0.001**
Chronic kidney	6 (1.59%)	16 (10%)	119 (22.49%)	38 (10.73%)	**<0.001**
Smoking	27 (7.14%)	12 (7.5%)	43 (8.13%)	25 (7.06%)	0.93
Other diseases	26 (6.88%)	9 (5.62%)	33 (6.24%)	36 (10.17%)	0.11
**Outcome**					
Pneumonia	172 (45.50%)	84 (52.5%)	282 (53.31%)	203 (57.34%)	**<0.05**
ICU	27 (7.14%)	16 (10%)	21 (3.97%)	20 (5.65%)	**<0.05**
Deaths	83 (21.96%)	48 (30%)	193 (36.48%)	134 (37.85%)	**<0.001**

Supplementary Table 6. Characterizing Risk Profiles in Hospitalized COVID-19 Men with Obesity on Validation sets: Insights from Validation Set Analysis of Selected Characteristics, Age Distribution, and Statistical Significance. We calculated the prevalence (as a percentage) of individuals exhibiting specific characteristics and determined the mean age (with standard error) within each risk profile. To assess statistical significance, we employed ANOVA and chi-squared tests for continuous and discrete variables, respectively, comparing differences between risk profiles.

**Feature**	**Non-Cardiometabolic (Low-Risk) (N=379)**	**Diabetic (High-Risk) (N=120)**	**Diabetic -Hypertensive (High-Risk) (N=429)**	**Hypertensive (High-Risk) (N=272)**	**p-value**
Age	51.14 (1)	60.43 (1.35)	65.32 (0.61)	64.8 (1.02)	**<0.001**
Diabetes	0	120 (100%)	429 (100%)	0	**<0.001**
COPD	15 (3.96%)	12 (10%)	43 (10.02%)	21 (7.72%)	**<0.05**
Asthma	14 (3.69%)	1 (0.83%)	9 (2.10%)	6 (2.21%)	0.26
Immunosuppression	17 (4.48%)	6 (5%)	17 (3.96%)	10 (3.68%)	0.92
Hypertension	0	0	429 (100%)	272 (100%)	**<0.001**
Cardiovascular	20 (5.28%)	11 (9.17%)	93 (21.68%)	52 (19.12%)	**<0.001**
Chronic kidney	16 (4.22%)	11 (9.17%)	104 (24.24%)	27 (9.93%)	**<0.001**
Smoking	66 (17.41%)	21 (17.5%)	98 (22.84%)	45 (16.54%)	0.11
Other diseases	25 (6.61%)	5 (4.17%)	22 (5.13%)	19 (6.98%)	0.57
**Outcome**					
Pneumonia	219 (57.78%)	75 (62.5%)	268 (62.47%)	155 (56.98%)	0.36
ICU	35 (9.23%)	15 (12.5%)	30 (6.99%)	24 (8.82%)	**<0.05**
Deaths	111 (29.29%)	44 (36.67%)	166 (38.69%)	106 (38.97%)	**<0.05**

Supplementary Table 7. Characterizing Risk Profiles in Hospitalized COVID-19 Women without Obesity on Validation sets: Insights from Validation Set Analysis of Selected Characteristics, Age Distribution, and Statistical Significance. We calculated the prevalence (as a percentage) of individuals exhibiting specific characteristics and determined the mean age (with standard error) within each risk profile. To assess statistical significance, we employed ANOVA and chi-squared tests for continuous and discrete variables, respectively, comparing differences between risk profiles.

**Feature**	**Pregnant (Low-Risk) (N=878)**	**Diverse Health (Low-Risk) (N=6254)**	**Diabetic (High-Risk) (N=830)**	**Hypertensive (High-Risk) (N=1532)**	**Diabetic-hypertensive (High-Risk) (N=1865)**	**p-value**
Age	27.01 (0.21)	44.55 (0.34)	62.02 (0.61)	69.87 (0.46)	68.81 (0.32)	**<0.001**
Pregnancy	878 (100%)	0	0	0	7 (0.37%)	**<0.001**
Diabetes	45 (5.12%)	0	830 (100%)	0	1865 (100%)	**<0.001**
COPD	0	126 (2.01%)	47 (5.66%)	130 (8.49%)	124 (6.65%)	**<0.001**
Asthma	15 (1.71%)	123 (1.97%)	21 (2.53%)	45 (2.94%)	38 (2.04%)	0.13
Immunosuppression	3 (0.34%)	250 (4.01%)	35 (4.22%)	58 (3.78%)	78 (4.18%)	**<0.001**
Hypertension	25 (2.85%)	0	0	1532 (100%)	1865 (100%)	**<0.001**
Cardiovascular	1 (0.11%)	136 (2.17%)	57 (6.87%)	148 (9.66%)	191 (10.24%)	**<0.001**
Chronic kidney	2 (0.23%)	158 (2.53%)	86 (10.36%)	178 (11.62%)	446 (23.91%)	**<0.001**
Smoking	9 (1.02%)	151 (2.41%)	27 (3.25%)	62 (4.05%)	85 (4.56%)	**<0.001**
Other diseases	36 (4.10%)	386 (6.17%)	36 (4.34%)	163 (10.64%)	117 (6.27%)	**<0.001**
**Outcome**						
Pneumonia	124 (14.12%)	1891 (30.24%)	380 (45.78%)	714 (46.61%)	918 (49.22%)	**<0.001**
ICU	20 (2.28%)	241 (3.85%)	46 (5.54%)	78 (5.09%)	106 (5.65%)	**<0.001**
Deaths	3 (0.34%)	1047 (16.74%)	256 (30.84%)	516 (33.68%)	674 (36.14%)	**<0.001**

Supplementary Table 8. Characterizing Risk Profiles in Hospitalized COVID-19 Men without Obesity on Validation sets: Insights from Validation Set Analysis of Selected Characteristics, Age Distribution, and Statistical Significance. We calculated the prevalence (as a percentage) of individuals exhibiting specific characteristics and determined the mean age (with standard error) within each risk profile. To assess statistical significance, we employed ANOVA and chi-squared tests for continuous and discrete variables, respectively, comparing differences between risk profiles.

**Feature**	**Non-Cardiometabolic (Low-Risk) (N=7464)**	**Diabetic (High-Risk) (N=1144)**	**Hypertensive (High-Risk) (N=1787)**	**Diabetic-Hypertensive (High-Risk) (N=2188)**	**p-value**
Age	47.84 (0.33)	64.97 (0.47)	69.63 (0.41)	69.05 (0.27)	**<0.001**
Diabetes	0	1144 (100%)	0	2188 (100%)	**<0.001**
COPD	222 (2.97%)	57 (4.98%)	151 (8.45%)	141 (6.44%)	**<0.001**
Asthma	108 (1.45%)	14 (1.22%)	28 (1.57%)	20 (0.91%)	0.22
Immunosuppression	326 (4.37%)	48 (4.19%)	59 (3.30%)	81 (3.70%)	0.16
Hypertension	0	0	1787 (100%)	2188 (100%)	**<0.001**
Cardiovascular	201 (2.69%)	57 (4.98%)	203 (11.36%)	244 (11.15%)	**<0.001**
Chronic kidney	201 (2.69%)	118 (10.31%)	298 (16.68%)	530 (24.22%)	**<0.001**
Smoking	444 (5.95%)	117 (10.23%)	183 (10.24%)	238 (10.88%)	**<0.001**
Other diseases	472 (6.32%)	58 (5.07%)	130 (7.27%)	130 (5.94%)	0.09
**Outcome**					
Pneumonia	2808 (37.62%)	604 (52.81%)	939 (52.55%)	1143 (52.24%)	**<0.001**
ICU	393 (5.26%)	86 (7.51%)	136 (7.61%)	140 (6.41%)	**<0.001**
Deaths	1917 (25.68%)	475 (41.52%)	746 (41.75%)	968 (44.24%)	**<0.001**

Supplementary Table 9. Comparing the Effect Sizes of Different Risk Profiles on the Validation Set of Hospitalized COVID-19 Women with Obesity on Validation sets. Significance levels (*, **, and ***) denote effect sizes, with *, **, and *** representing small (0.2-0.5 for Z, 1.5-2 for OR), medium (0.5-0.8 for Z, 2-3 for OR), and large (>0.8 for Z, >3 for OR) effect sizes, respectively.

**Effect Size**	**Non-CM/pregnant (Low-Risk) (N=378)**	**Diabetic (High-Risk) (N=160)**	**diabetic-hypertensive (High-Risk) (N=529)**	**hypertensive (High-Risk) (N=354)**
Age	**0.99**	0.01	**0.60**	**0.34**
Pregnancy	7.14 (4, 12.76)	0.64 (0.25, 1.62)	0.09 (0.03, 0.28)	0.55 (0.27, 1.14)
Diabetes	0	**Infinity**	**Infinity**	0
COPD	0.37 (0.21, 0.65)	0.79 (0.41, 1.5)	**1.56 (1.07, 2.27)**	1.4 (0.93, 2.11)
Asthma	0.92 (0.51, 1.63)	0.97 (0.43, 2.16)	1.08 (0.65, 1.81)	1 (0.56,1.79)
Immunosuppression	0.57 (0.29, 1.11)	1.52 (0.76,3.05)	0.9 (0.53, 1.53)	1.43 (0.83, 2.46)
Hypertension	0	0	**Infinity**	**Infinity**
Cardiovascular	0.3 (0.18, 0.49)	0.83 (0.49, 1.41)	**1.85 (1.34, 2.54)**	1.28 (0.9, 1.82)
Chronic kidney	0.08 (0.04, 0.18)	0.75 (0.44, 1.29)	**4.02 (2.89, 5.61)**	0.79 (0.54, 1.16)
Smoking	0.93 (0.59, 1.46)	1 (0.53, 1.86)	1.14 (0.77, 1.71)	0.91 (0.57, 1.45)
Other diseases	0.91 (0.58, 1.45)	0.73 (0.36, 1.48)	0.77 (0.5, 1.18)	**1.66 (1.09, 2.54)**
**Outcome**				
Pneumonia	0.7 (0.55, 0.88)	1.02 (0.73, 1.41)	1.08 (0.87, 1.34)	1.32 (1.04, 1.68)
ICU	1.33 (0.83, 2.13)	**1.95 (1.1, 3.45)**	0.54 (0.33, 0.9)	0.94 (0.56, 1.57)
Deaths	0.5 (0.38, 0.66)	1.13 (0.78, 1.62)	1.36 (1.08, 1.71)	1.4 (1.09, 1.8)

Supplementary Table 10. Comparing the Effect Sizes of Different Risk Profiles on the Validation Set of Hospitalized COVID-19 Men with Obesity on Validation sets. Significance levels (*, **, and ***) denote effect sizes, with *, **, and *** representing small (0.2-0.5 for Z, 1.5-2 for OR), medium (0.5-0.8 for Z, 2-3 for OR), and large (>0.8 for Z, >3 for OR) effect sizes, respectively.

**Feature**	**Non-Cardiometabolic (Low-Risk) (N=379)**	**Diabetic (High-Risk) (N=120)**	**Diabetic-Hypertensive (High-Risk) (N=429)**	**Hypertensive (High-Risk) (N=272)**
Age	**0.77**	0.01	**0.49**	**0.35**
Diabetes	0	**Infinity**	**Infinity**	0
COPD	0.4 (0.23, 0.71)	1.41 (0.74, 2.67)	**1.68 (1.09, 2.58)**	1.03 (0.62, 1.7)
Asthma	**1.93 (0.93, 4)**	0.3 (0.04, 2.26)	0.77 (0.35, 1.69)	0.85 (0.34, 2.1)
Immunosuppression	1.12 (0.62, 2.04)	1.24 (0.52, 2.97)	0.92 (0.51, 1.68)	0.85 (0.42, 1.72)
Hypertension	0	0	**Infinity**	**Infinity**
Cardiovascular	0.24 (1.15, 0.38)	0.56 (0.29, 1.06)	**2.29 (1.66, 3.17)**	**1.52 (1.07, 2.18)**
Chronic kidney	0.21 (0.12, 0.36)	0.64 (0.34, 1.22)	**4.25 (2.98, 6.05)**	0.67 (0.43, 1.04)
Smoking	0.84 (0.62, 1.16)	0.88 (0.54, 1.45)	1.43 (1.07, 1.92)	0.8 (0.56, 1.14)
Other diseases	1.19 (0.72, 1.97)	0.67 (0.26, 1.69)	0.8 (0.47, 1.34)	1.27 (0.73, 2.18)
**Outcome**				
Pneumonia	0.89 (0.69, 1.14)	1.14 (0.77, 1.68)	1.19 (0.94, 1.52)	0.86 (0.66, 1.13)
ICU	1.11 (0.72, 1.7)	**1.59 (0.89, 2.85)**	0.71 (0.46, 1.1)	1.03 (0.64, 1.65)
Deaths	0.66 (0.51, 0.86)	1.05 (0.71, 1.56)	1.23 (0.97, 1.58)	1.21 (0.91, 1.6)

Supplementary Table 11. Comparing the Effect Sizes of Different Risk Profiles on the Validation Set of Hospitalized COVID-19 Women without Obesity on Validation sets. Significance levels (*, **, and ***) denote effect sizes, with *, **, and *** representing small (0.2-0.5 for Z, 1.5-2 for OR), medium (0.5-0.8 for Z, 2-3 for OR), and large (>0.8 for Z, >3 for OR) effect sizes, respectively.

**Feature**	**Pregnant (Low-Risk) (N=878)**	**Diverse health (Low-Risk) (N=6254)**	**Diabetic (High-Risk) (N=830)**	**Hypertensive (High-Risk) (N=1532)**	**Diabetic-Hypertensive (High-Risk) (N=1865)**
Age	**1.44**	**0.67**	**0.49**	**0.94**	**0.96**
Pregnancy	**Infinity**	0	0	0	0.04 (0.02, 0.08)
Diabetes	0.16 (0.12,0.21)	0	**Infinity**	0	**Infinity**
COPD	0	0.28 (0.22, 0.34)	1.49 (1.09, 2.03)	**2.47 (2, 3.06)**	**1.98 (1.6, 2.46)**
Asthma	0.79 (0.46, 1.33)	0.84 (0.65, 1.08)	1.21 (0.77, 1.9)	1.48 (1.07, 2.05)	0.95 (0.67, 1.34)
Immunosuppression	0.08 (0.03, 0.26)	1.18 (0.97, 1.44)	1.15 (0.81, 1.63)	1.02 (0.77, 1.35)	1.15 (0.9, 1.48)
Hypertension	0.06 (0.04, 0.09)	0	0	**Infinity**	**Infinity**
Cardiovascular	0.02 (0, 0.15)	0.26 (0.22, 0.32)	**1.56 (1.17, 2.07)**	**2.62 (2.15, 3.2)**	**3.05 (2.54, 3.67)**
Chronic kidney	0.03 (0.01, 0.1)	0.16 (0.13,0.19)	1.44 (1.14, 1.82)	**1.74 (1.46, 2.07)**	**6.72 (5.82, 7.77)**
Smoking	0.32 (0.17, 0.63)	0.67 (0.53, 0.83)	1.12 (0.75, 1.67)	1.48 (1.12, 1.69)	**1.77 (1.38, 2.28)**
Other diseases	0.6 (0.42, 0.84)	0.89 (0.76, 1.03)	0.63 (0.45, 0.89)	**1.92 (1.6, 2.3)**	0.96 (0.78, 1.17)
**Outcome**					
Pneumonia	0.28 (0.23, 0.34)	0.6 (0.56, 0.65)	**1.59 (1.38, 1.84)**	**1.72 (1.54, 1.91)**	**1.99 (1.8, 2.2)**
ICU	0.5 (0.31, 0.78)	0.98 (0.77, 1.32)	1.33 (0.97, 1.82)	1.22 (0.95, 1.57)	1.43 (1.14, 1.78)
Deaths	0.01 (0, 0.03)	0.51 (0.46, 0.56)	**1.65 (1.41, 1.93)**	**2.01 (1.79, 2.26)**	**2.38 (2.14, 2.65)**

Supplementary Table 12. Comparing the Effect Sizes of Different Risk Profiles on the Validation Set of Hospitalized COVID-19 Men without Obesity on Validation sets. Significance levels (*, **, and ***) denote effect sizes, with *, **, and *** representing small (0.2-0.5 for Z, 1.5-2 for OR), medium (0.5-0.8 for Z, 2-3 for OR), and large (>0.8 for Z, >3 for OR) effect sizes, respectively.

**Feature**	**Non-Cardiometabolic (Low-Risk) (N=7464)**	**Diabetic (High-Risk) (N=1144)**	**Hypertensive (High-Risk) (N=1787)**	**Diabetic-hypertensive (High-Risk) (N=2188)**
Age	**0.90**	**0.44**	**0.7**	**0.74**
Diabetes	0	**Infinity**	0	**Infinity**
COPD	0.42<br>(0.35, 0.5)	0.74<br>(0.56, 0.98)	**2.28<br>(1.88, 2.77)**	**1.6<br>(1.31, 194)**
Asthma	1.2<br>(0.87, 1.64)	0.9<br>(0.52, 1.55)	1.19<br>(0.79, 1.8)	0.63<br>(0.39, 1.01)
Immunosuppression	1.2<br>(1, 1.44)	1.03<br>(0.76, 1.4)	0.78<br>(0.59, 1.02)	0.88<br>(0.69, 1.13)
Hypertension	0	0	**Infinity**	**Infinity**
Cardiovascular	0.25<br>(0.21, 0.3)	0.96<br>(0.73, 1.27)	**2.63<br>(2.21, 3.12)**	**2.7<br>(2.3, 3.18)**
Chronic kidney	0.12<br>(0.1, 0.14)	1.16<br>(0.95, 1.42)	**2.21<br>(1.92, 2.55)**	**5.06<br>(4.46, 5.75)**
Smoking	0.54<br>(0.47, 0.61)	1.39<br>(1.14, 1.71)	1.43<br>(1.21, 1.69)	**1.58<br>(1.36, 1.85)**
Other diseases	1.02<br>(0.88, 1.18)	0.78<br>(0.59, 1.03)	1.2<br>(0.99, 1.46)	0.93<br>(0.77, 1.13)
**Outcome**				
Pneumonia	0.55<br>(0.51, 0.59)	**1.5<br>(1.33, 1.69)**	**1.52<br>(1.37, 1.68)**	**1.52<br>(1.39, 1/67)**
ICU	0.69<br>(0.6, 0.81)	1.35<br>(1.07, 1.71)	1.4<br>(1.16, 1.7)	1.13<br>(0.93, 1.36)
Deaths	0.46<br>(0.43, 0.5)	**1.53<br>(1.35, 1.73)**	**1.59<br>(1.43, 1.76)**	**1.83<br>(1.67, 2.02)**

Supplementary Table 13. Comparing of pairwise P-Values for Features and Outcomes in Discovered Risk Profiles for Hospitalized COVID-19 Women with Obesity on Validation sets. Note: In the table, 1 indicates a statistically significant difference (p-value < 0.05), while 0 suggests no significant difference (p-value > 0.05).

Features	Non-CM/pregnant vs diabetic	Non-CM/pregnant vs diabetic-hypertensive	Non-CM/pregnant vs hypertensive	diabetic vs diabetic-hypertensive	diabetic vs hypertensive	diabetic-hypertensive vs hypertensive
Age	1	1	1	1	1	0
Pregnancy	1	1	1	1	0	1
Diabetes	1	1	0	0	1	1
COPD	0	1	1	0	0	0
Asthma	0	0	0	0	0	0
Immunosuppression	0	0	0	0	0	0
Hypertension	0	1	1	1	1	0
Cardiovascular	1	1	1	0	0	0
Chronic kidney	1	1	1	1	0	1
Smoking	0	0	0	0	0	0
Other diseases	0	0	0	0	0	1
**Outcome**						
Pneumonia	0	1	1	0	0	0
ICU	0	1	1	1	0	0
Deaths	0	1	1	0	0	0

Supplementary Table 14. Comparing of pairwise P-Values for Features and Outcomes in Discovered Risk Profiles for Hospitalized COVID-19 Men with Obesity on Validation sets. Note: In the table, 1 indicates a statistically significant difference (p-value < 0.05), while 0 suggests no significant difference (p-value > 0.05).

**Features**	**Non-Cardiometabolic vs diabetic**	**Non-Cardiometabolic vs diabetic-hypertensive**	**Non-Cardiometabolic vs hypertensive**	**diabetic vs diabetic-hypertensive**	**diabetic vs hypertensive**	**diabetic-hypertensive vs hypertensive**
Age	1	1	1	1	1	0
Diabetes	1	1	0	0	1	1
COPD	1	1	0	0	0	0
Asthma	0	0	0	0	0	0
Immunosuppression	0	0	0	0	0	0
Hypertension	0	1	1	1	1	0
Cardiovascular	0	1	1	1	1	0
Chronic kidney	0	1	1	1	0	1
Smoking	0	0	0	0	0	0
Other diseases	0	0	0	0	0	0
**Outcome**						
Pneumonia	0	0	0	0	0	0
ICU	0	0	0	0	0	0
Deaths	0	1	1	0	0	0

Supplementary Table 15. Comparing of pairwise P-Values for Features and Outcomes in Discovered Risk Profiles for Hospitalized COVID-19 Women without Obesity on Validation sets. Note: In the table, 1 indicates a statistically significant difference (p-value < 0.05), while 0 suggests no significant difference (p-value > 0.05).

**Features**	**Pregnant vs Diverse Health issues**	**Pregnant vs diabetic**	**Pregnant vs hypertensive**	**Pregnant vs diabetic-hypertensive**	**Diverse Health vs diabetic**	**Diverse Health vs hypertensive**
Age	1	1	1	1	1	1
Pregnancy	1	1	1	1	0	0
Diabetes	1	1	1	1	1	0
COPD	1	1	1	1	1	1
Asthma	0	0	0	0	0	1
Immunosuppression	1	1	1	1	0	0
Hypertension	1	1	1	1	0	1
Cardiovascular	1	1	1	1	1	1
Chronic kidney	1	1	1	1	1	1
Smoking	1	1	1	1	0	1
Other diseases	1	0	1	1	1	1
**Outcome**						
Pneumonia	1	1	1	1	1	1
ICU	1	1	1	1	1	1
Deaths	1	1	1	1	1	1

**Features**	**Diverse Health vs diabetic - hypertensive**	**Diabetic vs hypertensive**	**Diabetic vs diabetic - hypertensive**	**hypertensive vs diabetic - hypertensive**
Age	1	1	1	0
Pregnancy	1	0	0	1
Diabetes	1	1	0	1
COPD	1	1	0	1
Asthma	0	0	0	0
Immunosuppression	0	0	0	0
Hypertension	1	1	1	0
Cardiovascular	1	1	1	0
Chronic kidney	1	0	1	1
Smoking	1	0	0	0
Other diseases	0	1	0	1
**Outcome**				
Pneumonia	1	0	0	0
ICU	1	0	0	0
Deaths	1	0	1	0

Supplementary Table 16. Comparing of pairwise P-Values for Features and Outcomes in Discovered Risk Profiles for Hospitalized COVID-19 Men without Obesity on Validation sets. Note: In the table, 1 indicates a statistically significant difference (p-value < 0.05), while 0 suggests no significant difference (p-value > 0.05).

**Features**	**Non-Cardiometabolic vs diabetic**	**Non-Cardiometabolic vs hypertensive**	**Non-Cardiometabolic vs diabetic-hypertensive**	**diabetic vs hypertensive**	**diabetic vs diabetic-hypertensive**	**hypertensive vs diabetic-hypertensive**
Age	1	1	1	1	1	0
Diabetes	1	0	1	1	0	1
COPD	1	1	1	1	0	1
Asthma	0	0	0	0	0	0
Immunosuppression	0	1	0	0	0	0
Hypertension	0	1	1	1	1	0
Cardiovascular	1	1	1	1	1	0
Chronic kidney	1	1	1	1	1	1
Smoking	1	1	1	0	0	0
Other diseases	0	0	0	1	0	0
**Outcome**						
Pneumonia	1	1	1	0	0	0
ICU	1	1	1	0	0	0
Deaths	1	1	1	0	0	0

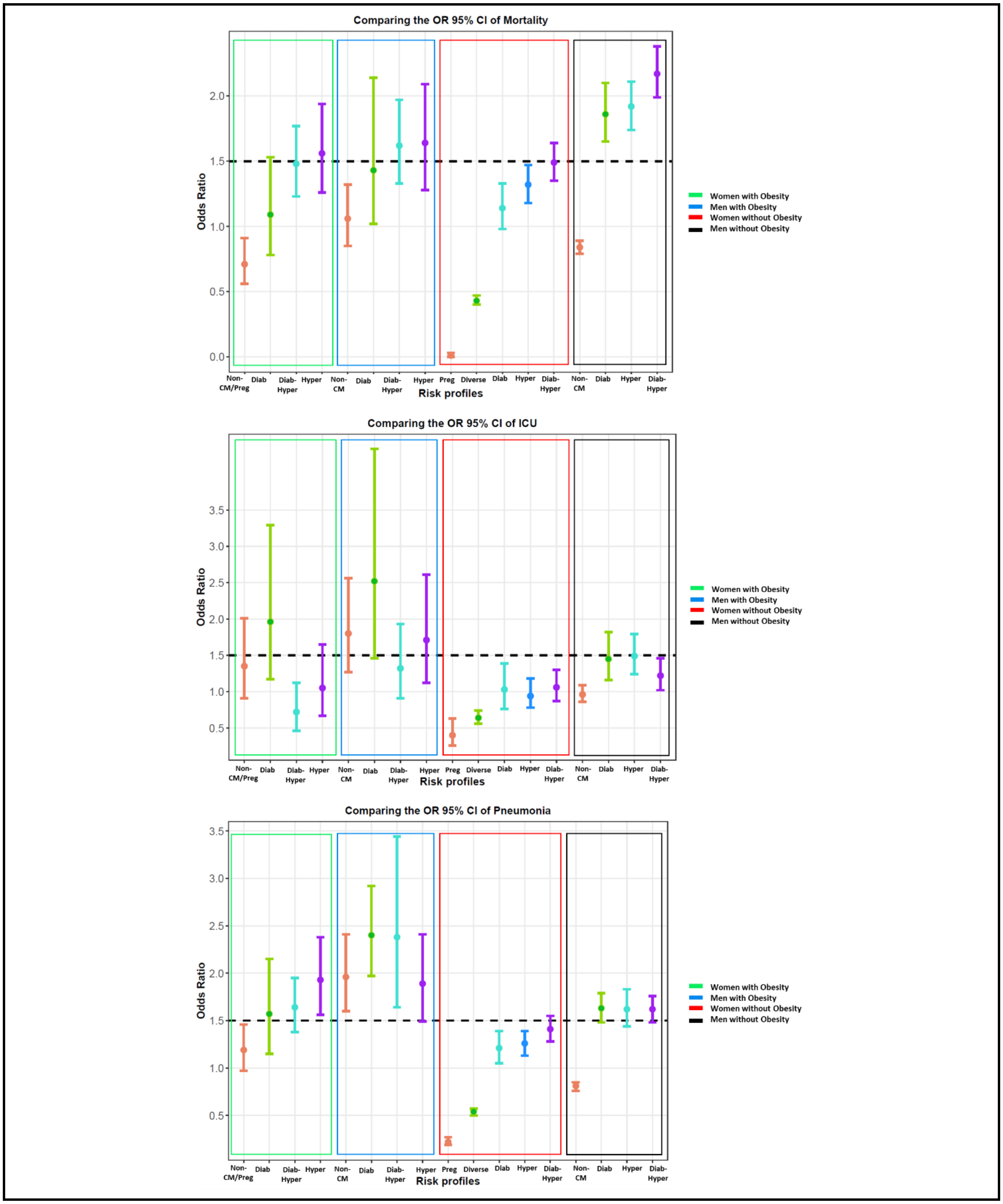

Supplementary Figure 7. Comparison of Odds Ratios (95% Confidence Intervals) of the outcomes (the rate of Mortality, ICU and Pneumonia) among Four Categories on Validation sets: Women with and without obesity, Men with and without obesity, for Discovered Risk profiles.

Supplementary Table 17. The distribution of patients among risk profiles and across different states in the validation set of hospitalized COVID-19 Women with obesity.

States	Diabetic-Hypertensive profile	Hypertensive profile	Diabetic profile	Non-CM/Pregnant profile
AGUASCALIENTES	**40 (47.06%)**	22 (25.88%)	10 (11.76%)	13 (15.29%)
BAJA CALIFORNI	**113 (43.63%)**	73 (28.18%)	16 (6.18%)	57 (22.01%)
BAJA CALIFORNIA SUR	18 (30%)	17 (28.3%)	5 (8.33%)	**20 (33.3%)**
CAMPECHE	10 (30.30%)	**11 (33.33%)**	2 (6.06%)	10 (30.30%)
COAHUILA DE ZARAGOZA	**37 (41.11%)**	21 (23.33%)	13 (14.44%)	19 (21.11%)
COLIMA	**23 (43.40%)**	12 (22.64%)	5 (9.43%)	13 (24.53%)
CHIAPAS	**24 (48.98%)**	13 (26.53%)	3 (6.12%)	9 (18.37%)
CHIHUAHUA	**64 (39.75%)**	41 (25.46%)	21 (13.04%)	35 (21.74%)
CIUDAD DE MÉXICO	**189 (31.55%)**	155 (25.88%)	69 (11.52%)	186 (31.05%)
DURANGO	**37 (52.11%)**	15 (21.13%)	8 (11.27%)	11 (15.49%)
GUANAJUATO	**139 (41.87%)**	71 (21.38%)	33 (9.94%)	89 (26.80%)
GUERRERO	24 (36.92%)	7 (10.77%)	8 (12.31%)	**26 (0.4%)**
HIDALGO	38 (32.48%)	33 (28.2%)	7 (5.98%)	**39 (33.33%)**
JALISCO	**169 (39.30%)**	109 (25.35%)	43 (10%)	109 (25.34%)
MÉXICO	130 (32.01%)	90 (22.17%)	49 (12.07%)	**137 (33.74%)**
MICHOACÁN DE OCAMPO	**38 (32.76%)**	36 (31.03%)	10 (8.62%)	32 (27.59%)
MORELOS	**24 (40%)**	14 (23.33%)	5 (8.33%)	17 (28.33%)
NAYARIT	**16 (39.02%)**	9 (21.95%)	2 (4.88%)	14 (34.14%)
NUEVO LEÓN	62 (33.51%)	35 (18.92%)	23 (12.43%)	**65 (35.13%)**
OAXACA	**37 (39.36%)**	22 (23.40%)	14 (14.89%)	21 (22.34%)
PUEBLA	**63 (37.06%)**	33 (19.41%)	26 (15.29%)	48 (38.23%)
QUERÉTARO	**46 (44.66%)**	21 (20.39%)	8 (7.77%)	28 (27.18%)
QUINTANA ROO	14 (28.57%)	10 (20.41%)	9 (18.37%)	**16 (32.65%)**
SAN LUIS POTOSÍ	**45 (38.13%)**	27 (22.88%)	11 (9.32%)	35 (29.66%)
SINALOA	**67 (42.95%)**	44 (28.20%)	15 (9.61%)	30 (19.23%)
SONORA	**58 (34.73%)**	47 (28.14%)	19 (11.38%)	43 (25.75%)
TABASCO	22 (37.29%)	10 (16.95%)	3 (5.08%)	**24 (40.68%)**
TAMAULIPAS	**96 (46.38%)**	40 (19.32%)	25 (12.08%)	46 (22.22%)
TLAXCALA	**17 (43.59%)**	3 (7.69%)	4 (10.25%)	15 (38.46%)
VERACRUZ DE IGNACIO DE LA LLAVE	66 (34.37%)	37 (19.27%)	22 (11.46%)	**67 (34.89%)**
YUCATÁN	32 (32.32%)	23 (23.23%)	10 (10.10%)	**34 (34.34%)**
ZACATECAS	**28 (39.44%)**	23 (32.39%)	6 (8.45%)	14 (19.72%)

Supplementary Table 18. The distribution of patients among risk profiles and across different states in the validation set of hospitalized COVID-19 Men with obesity.

States	Diabetic-Hypertensive profile	Hypertensive profile	Diabetic profile	Non-Cardiometabolic profile
AGUASCALIENTES	23 (31.51%)	20 (27.40%)	4 (5.48%)	**26 (35.62%)**
BAJA CALIFORNI	**107 (43.85%)**	63 (25.82%)	13 (5.33%)	61 (25%)
BAJA CALIFORNIA SUR	27 (41.54%)	15 (23.08%)	7 (10.77%)	**16 (24.61%)**
CAMPECHE	**12 (30%)**	10 (25%)	8 (20%)	10 (25%)
COAHUILA DE ZARAGOZA	**26 (35.13%)**	17 (22.97%)	9 (12.16%)	22 (29.73%)
COLIMA	**21 (51.22%)**	6 (14.63%)	3 (7.32%)	11 (26.83%)
CHIAPAS	**13 (35.13%)**	11 (29.73%)	1 (2.7%)	12 (32.43%)
CHIHUAHUA	**41 (33.33%)**	34 (27.64%)	13 (10.57%)	35 (28.45%)
CIUDAD DE MÉXICO	157 (31.21%)	131 (26.04%)	45 (8.95%)	**170 (33.8%)**
DURANGO	8 (23.53%)	**12 (35.29%)**	4 (11.76%)	10 (29.41%)
GUANAJUATO	**99 (37.93%)**	54 (20.69%)	35 (13.41%)	73 (27.97%)
GUERRERO	17 (29.82%)	13 (22.81%)	8 (14.03%)	**19 (33.33%)**
HIDALGO	44 (34.37%)	21 (16.41%)	17 (13.28%)	**46 (35.94%)**
JALISCO	**137 (40.17%)**	80 (23.46%)	25 (7.33%)	99 (29.03%)
MÉXICO	108 (33.23%)	59 (18.15%)	33 (10.15%)	**125 (38.46%)**
MICHOACÁN DE OCAMPO	**38 (41.30%)**	16 (17.39%)	7 (7.61%)	31 (33.69%)
MORELOS	20 (28.57%)	19 (27.14%)	5 (7.14%)	**26 (37.14%)**
NAYARIT	10 (21.74%)	13 (28.26%)	4 (8.69%)	**19 (41.30%)**
NUEVO LEÓN	**59 (35.54%)**	27 (16.26%)	26 (15.66%)	54 (32.53%)
OAXACA	25 (28.73%)	13 (14.94%)	15 (17.24%)	**34 (39.08%)**
PUEBLA	**49 (35.77%)**	27 (19.71%)	14 (10.22%)	47 (34.31%)
QUERÉTARO	32 (32.99%)	21 (21.65%)	8 (8.25%)	**36 (37.11%)**
QUINTANA ROO	**14 (42.42%)**	9 (27.27%)	5 (15.15%)	5 (15.15%)
SAN LUIS POTOSÍ	**27 (32.93%)**	23 (28.05%)	9 (10.98%)	23 (28.05%)
SINALOA	**49 (40.83%)**	35 (29.17%)	6 (5%)	30 (25%)
SONORA	**48 (36.64%)**	34 (25.95%)	16 (12.21%)	33 (25.19%)
TABASCO	16 (27.12%)	14 (23.73%)	8 (13.56%)	**21 (35.59%)**
TAMAULIPAS	**64 (47.76%)**	25 (18.66%)	12 (8.95%)	33 (24.63%)
TLAXCALA	9 (31.03%)	2 (6.9%)	4 (13.79%)	**14 (48.27%)**
VERACRUZ DE IGNACIO DE LA LLAVE	**77 (38.69%)**	45 (22.61%)	21 (10.55%)	56 (28.14%)
YUCATÁN	26 (28.89%)	24 (26.67%)	6 (6.67%)	**34 (37.78%)**
ZACATECAS	**35 (43.75%)**	21 (26.25%)	9 (11.25%)	15 (18.75%)
